# IL-38 Aggravates Atopic Dermatitis via Facilitating Migration of Langerhans cells

**DOI:** 10.7150/ijbs.93843

**Published:** 2024-05-27

**Authors:** Chengcheng Yue, Yawen Hu, Jiadong Yu, Hong Zhou, Pei Zhou, Jing Hu, Xiaoyan Wang, Linna Gu, Ya Li, Yuting Feng, Fanlian Zeng, Fulei Zhao, Guolin Li, Qixiang Zhao, Chen Zhang, Huaping Zheng, Wenling Wu, Xinai Cui, Nongyu Huang, Zhen Wang, Kaijun Cui, Jiong Li

**Affiliations:** 1 State Key Laboratory of Biotherapy and Cancer Center, West China Hospital, Sichuan University, and Collaborative Innovation Center for Biotherapy, 1 Keyuan 4th Road, Gaopeng Street, Chengdu, Sichuan 610041, China.; 2 Department of Liver Surgery & Liver Transplantation, West China Hospital, Sichuan University and Collaborative Innovation Center of Biotherapy, 37 Guo Xue Road, Chengdu, Sichuan 610041, China.; 3 Department of Cardiology, West China Hospital, Sichuan University, 37 Guoxue Road, Chengdu, Sichuan 610041, China.; 4 CDUTCM-KEELE Joint Health and Medical Sciences Institute, Chengdu University of Traditional Chinese Medicine, Chengdu, 611137, China.

**Keywords:** Atopic dermatitis, Interleukin-38, Langerhans cells migrating

## Abstract

Atopic dermatitis (AD) is a common inflammation skin disease that involves dysregulated interplay between immune cells and keratinocytes. Interleukin-38 (IL-38), a poorly characterized IL-1 family cytokine, its role and mechanism in the pathogenesis of AD is elusive. Here, we show that IL-38 is mainly secreted by epidermal keratinocytes and highly expressed in the skin and downregulated in AD lesions. We generated IL-38 keratinocyte-specific knockout mice (*K14^Cre/+^-IL-38^f/f^*) and induced AD models by 2,4-dinitrofluorobenzene (DNFB). Unexpectedly, after treatment with DNFB, *K14^Cre/+^-IL-38^f/f^* mice were less susceptible to cutaneous inflammation of AD. Moreover, keratinocyte-specific deletion of IL-38 suppressed the migration of Langerhans cells (LCs) into lymph nodes which results in disturbed differentiation of CD4^+^T cells and decreased the infiltration of immune cells into AD lesions. LCs are a type of dendritic cell that reside specifically in the epidermis and regulate immune responses. We developed LC-like cells *in vitro* from mouse bone marrow (BM) and treated with recombined IL-38. The results show that IL-38 depended on IL-36R, activated the phosphorylated expression of IRAK4 and NF-κB P65 and upregulated the expression of CCR7 to promoting the migration of LCs, nevertheless, the upregulation disappeared with the addition of IL-36 receptor antagonist (IL-36RA), IRAK4 or NF-κB P65 inhibitor. Furthermore, after treatment with IRAK4 inhibitors, the experimental AD phenotypes were alleviated and so IRAK4 is considered a promising target for the treatment of inflammatory diseases. Overall, our findings indicated a potential pathway that IL-38 depends on IL-36R, leading to LCs migration to promote AD by upregulating CCR7 via IRAK4/NF-κB and implied the prevention and treatment of AD, supporting potential clinical utilization of IRAK4 inhibitors in AD treatment.

## Introduction

Atopic dermatitis (AD) is a common inflammatory dermatosis that affects individuals of all ages and ethnicities 1, it leads to an incessant itching cycle, significantly impacting quality of life 2. Notably, a recent investigation assessing the impact of AD using disability-adjusted life-years (DALYs) revealed it imposes the greatest burden among dermatological disorders and ranks 15th among non-fatal illnesses 3. With increasing data on epidemiology, pathophysiology, and therapy, progress in AD research remains strong and still full of challenges 4.

AD is closely associated with impairment of epidermal function and immune cell disorders, as well as lifestyle and environmental factors 5, 6. Immune cell dynamics within the skin assume a pivotal role in the pathogenesis of AD. In current opinion, AD is an inflammatory skin disease driven by Th2^7^. Langerhans cells (LCs) are the resident antigen-presenting cells in the epidermis and migratory LCs can quickly migrate to skin-draining lymph nodes following inflammatory stimulation, playing a crucial role in the development of AD 8, 9. When the skin barrier is compromised, allergens enter the skin to trigger the production of pro-inflammatory cytokines like TSLP by keratinocytes, which prompts the activation of inflammatory cells and their recruitment to the AD-lesions 10. LCs process antigens, migrate to lymph nodes, and promote differentiation of naive CD4^+^ T cells into Th2 cells which circulate and infiltrate the lesions, amplifying the Th2-driven inflammation 11. Moreover, facilitating B cell to produce IgE, activates mast cells, enhancing Th2 cell migration and release of inflammatory cytokines, exacerbating AD pathogenesis 12, 13. Despite the costly and limited availability of drugs for AD therapy 14, research on AD pathogenesis remains an important and meaningful topic.

In recent years, the IL-1 family, particularly the IL-36 subfamily, has emerged as a key regulator of inflammatory diseases, including skin disorders 15. Among them, Interleukin-38 (IL-38) is a newly discovered member with limited understanding of its structure, expression regulation, and signaling pathways 16, 17. The IL-38 gene has five exons and is located on human chromosome 2q13-14.1^18^. It was reported that IL-38 is expressed in basal epithelial cells as a primary source in humans and keratinocytes for mice 19. IL-38 exerts immunoregulatory activities in multi-type inflammatory diseases and some studies suggest IL-38 acts as an antagonist by binding to IL-36R, while recent findings indicate that IL-38 can also bind to IL-36R, IL1RAPL1 or IL-1R and induce the production of pro-inflammatory cytokines in response to various stimuli 20-23. Extensive research has linked IL-38 to several diseases, including psoriasis 24, psoriatic arthritis 25, ankylosing spondylitis 26, systemic lupus erythematosus 27, and experimental autoimmune encephalomyelitis 28, however, its relevance in AD remains unexplored.

Here, we verify the potential role of IL-38 in AD from the IL-38 perspective, for the first time. Our study demonstrate that IL-38 depends on IL-36R activates the IRAK4/NF-κB signaling pathway, which in turn up-regulates CCR7, facilitating the migration of LCs, in thus promotes the development of DNFB-induced AD-like skin inflammation. While the DNFB-induced AD-like skin inflammation was attuned after treated with the inhibitor of IRAK4. Overall, our findings facilitate the understanding the pathogenesis of AD, implying the role of IRAK4 inhibitor in AD and offering potential new preventive and therapeutic strategy as well as the novel insights of new medications in treating AD.

## Results

### IL-38 is abundantly expressed in skin and closely related to the occurrence and development of AD

**Figure 1:**
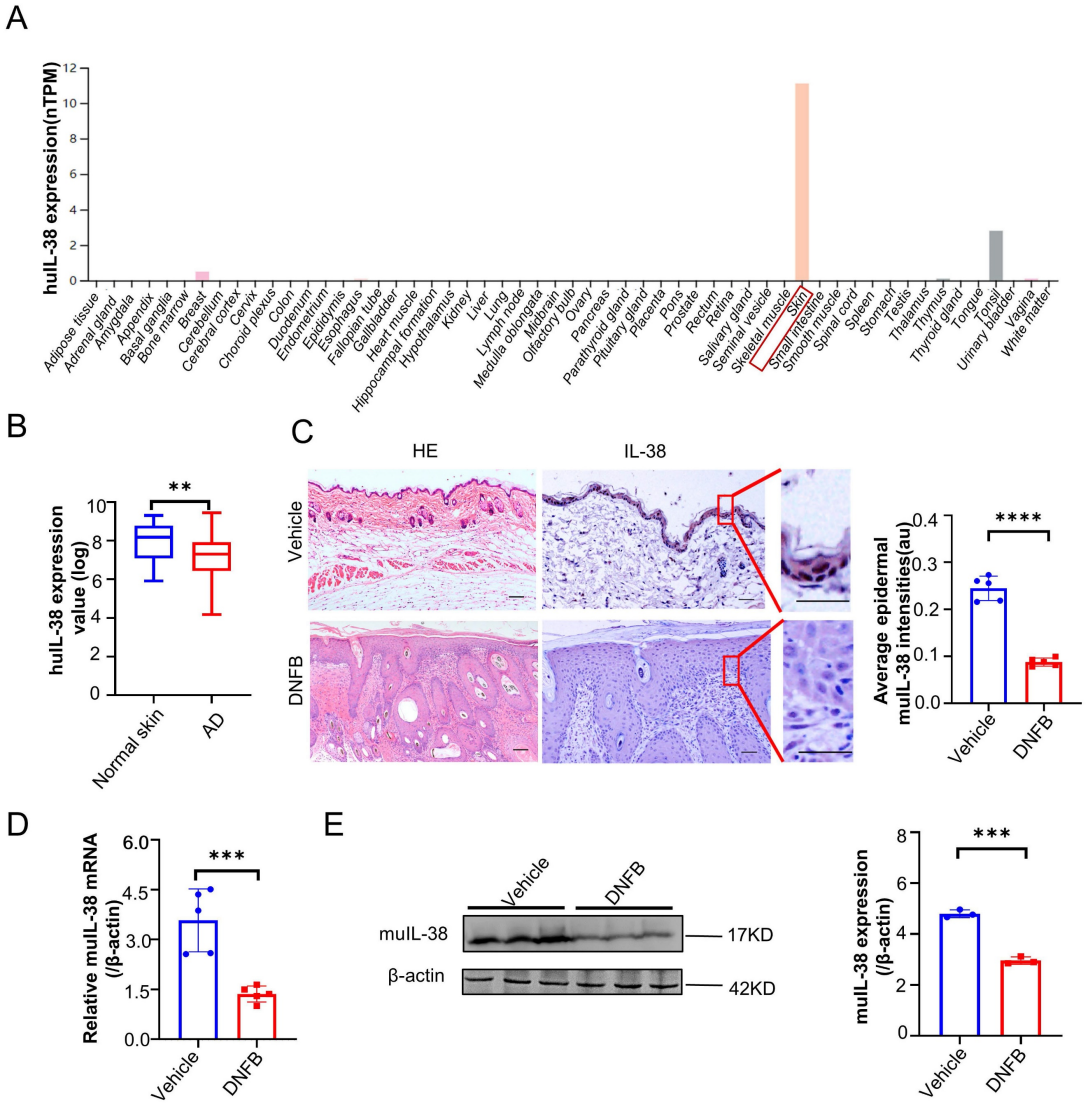
** IL-38 is closely associated with the occurrence and development of atopic dermatitis.** (A) IL-38 expression levels in normal human tissues from the Human Protein Atlas database (huIL-38, Human IL-38). (B) Relative expression of IL-38 in human normal skin (n=22) and lesions of AD patients (n=54) were analyzed by GEO Datasets (GSE140684). (C) Representative micrographs of mouse normal skin (n=5) and AD-lesion (n=5) section stained with hematoxylin-eosin (H&E) (left) and anti-IL-38 antibody (right). Scale bars represent 100 μm. The graph shows the quantification of mean IL-38 expression per high-powered field in tissues. (D) Relative expression of IL-38 in mouse normal skin (n=5) and DNFB induced AD-lesion (n=5) was quantified by RT-qPCR. (E) Representative western blot brands of IL-38 in mouse normal skin (n=3) and DNFB induced AD-lesion (n=3). The graph shows the quantification of mean IL-38 expression in tissues. Error bars represent the mean ± SD. *p < 0.05; **p < 0.01; ***p < 0.001; ****P<0.0001; p values were calculated using Student's t test.

To investigate the potential role of IL-38 in the development of AD, we analyzed IL-38 mRNA expression levels in different tissues using the Human Protein Atlas database (HPA). Our analysis revealed high expression of IL-38 in the skin (Figure 1A), consistent with previous reports 29. This finding suggests that IL-38 may play a significant role in skin-related diseases. To explore the possible association between IL-38 and AD, we analyzed data from Gene Expression Omnibus Dataset (GEO) and found that IL-38 expression was downregulated in AD-lesions compared to normal skin tissues (Figure 1B). To further investigate this correlation, we used a suitable animal model of AD-like inflammation in mice by 2,4-dinitrofluorobenzene (DNFB) inducing, which can induce inflammation similar to clinical AD and lead to a series of immune responses after entering the skin 30, 31. We referred to the literature 32, 33 and constructed the AD model (Figure S1A-B). The AD model showed a significant increase in Th2 and Th1 cells in the lymph nodes (Figure S1C), elevated expression of IL-4, IFN-γ, TSLP cytokines in the lesions (Figure S1D), and increased serum IgE levels (Figure S1E), consistent with previous findings 34, indicating the successfully construction of the DNFB-induced AD model. Immunohistochemistry, RT-qPCR, and Western blot analysis confirmed downregulated expression of IL-38 in the skin lesions of the AD model (Figure 1C-E), consistent with the findings in AD patients (Figure 1B). These results indicate the potential involvement of IL-38 in the occurrence and development of AD.

### IL-38 keratinocyte-specific knockout has no impact on skin barrier and immunity in mice at homeostasis

**Figure 2:**
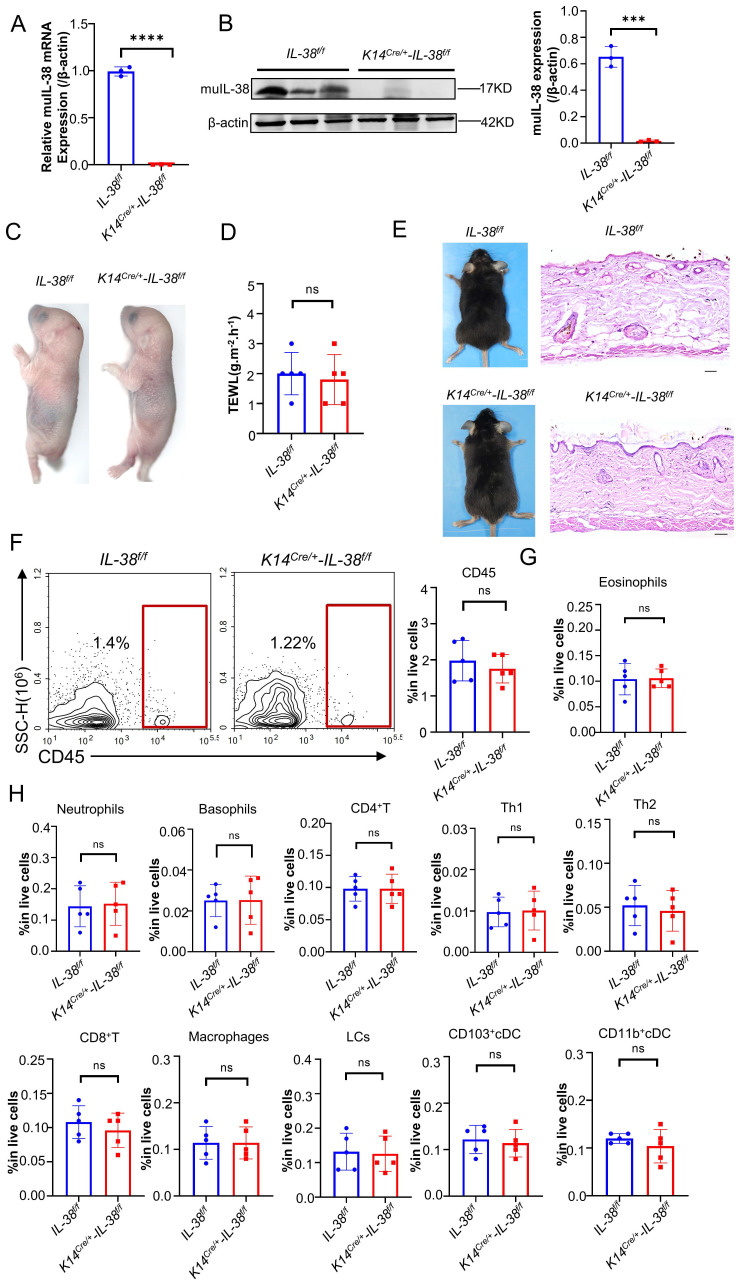
** IL-38 keratinocyte-specific knockout mice were successfully constructed and the keratinocyte-specific deletion of IL-38 had no significant influence on the skin barrier and immunity of mice.** (A) Relative expression of IL-38 in* K14^Cre/+^-IL-38^f/f^* (n=3) and* IL-38^f/f^* mouse(n=3) of skin was quantified by RT-qPCR. (B) Representative western blot brands of IL-38 in* K14^Cre/+^-IL-38^f/f^* (n=3) and* IL-38^f/f^* mouse(n=3) of skin epidermis. The graph shows the quantification of mean IL-38 expression in tissues. (C) Representative graph of skin barrier-dependent dye exclusion assay using toluidine blue in *K14^Cre/+^-IL-38^f/f^* and* IL-38^f/f^* fetus mouse. (D) TEWL assay measured on ventral surface of *K14^Cre/+^-IL-38^f/f^* (n=5) and* IL-38^f/f^* (n=5) fetus mouse. (E) Representative graph of *K14^Cre/+^-IL-38^f/f^* and* IL-38^f/f^* mouse appearance(left) and skin with H&E staining(right). Scale bars represent 100 μm. (F) Flow cytometry detection number of CD45 cells in *K14^Cre/+^-IL-38^f/f^* (n=5) and* IL-38^f/f^* (n=5) mice of skin. (G and H) Flow cytometry assay number of immune cells in *K14^Cre/+^-IL-38^f/f^* (n=5) and* IL-38^f/f^* (n=5) mice of skin. Error bars represent the mean ± SD. ns, not significant; ***p < 0.001; ****P<0.0001; p values were calculated using Student's t test.

IL-38 exhibits high expression in the skin and is predominantly secreted by keratinocytes. To further elucidate the role and mechanisms of IL-38 in AD, we generated *IL-38^f/f^* mice through transgenic insertion of the Floxp site, and obtained IL-38 keratinocyte-specific knockout mice (*K14^Cre/+^-IL-38^f/f^*) by crossing with K14-promoter Cre (*Krt14-Cre*) transgenic mice (Figure S2A-C), as we previously reported 22. RT-qPCR and Western blot analyses confirmed the specific knockout of IL-38 in *K14^Cre/+^-IL-38^f/f^* mice (Figure 2 A-B). These findings affirm the successful generation of IL-38 keratinocyte-specific knockout mice.

Disruption of certain proteins in skin keratinocytes through gene knockout may lead to the spontaneous development of skin diseases in mice, highlighting the crucial role of the intact epidermis in maintaining a protective barrier function, preventing water loss, and safeguarding against the entry of foreign substances and microorganisms 35. Therefore, to investigate whether skin-specific knockout of IL-38 affects skin barrier function in mice, we performed toluidine blue staining (Figure 2C) and skin transdermal water loss assay (TEWL) (Figure 2D) to assess skin barrier function in fetal mice. Our findings revealed no significant difference in skin barrier function following IL-38 knockout. We examined the apparent and epidermal thickness as well as local immunity of knockout and control mice at 6-8 weeks of age, showing no notable differences between the two groups (Figure 2E-H). These results indicate that the specific knockout of IL-38 doesn't cause discernible physiological changes in the mice under steady-state conditions, thereby validating its suitability for subsequent research.

### IL-38 keratinocyte-specific deletion suppressed the DNFB-induced AD-like skin inflammation

**Figure 3:**
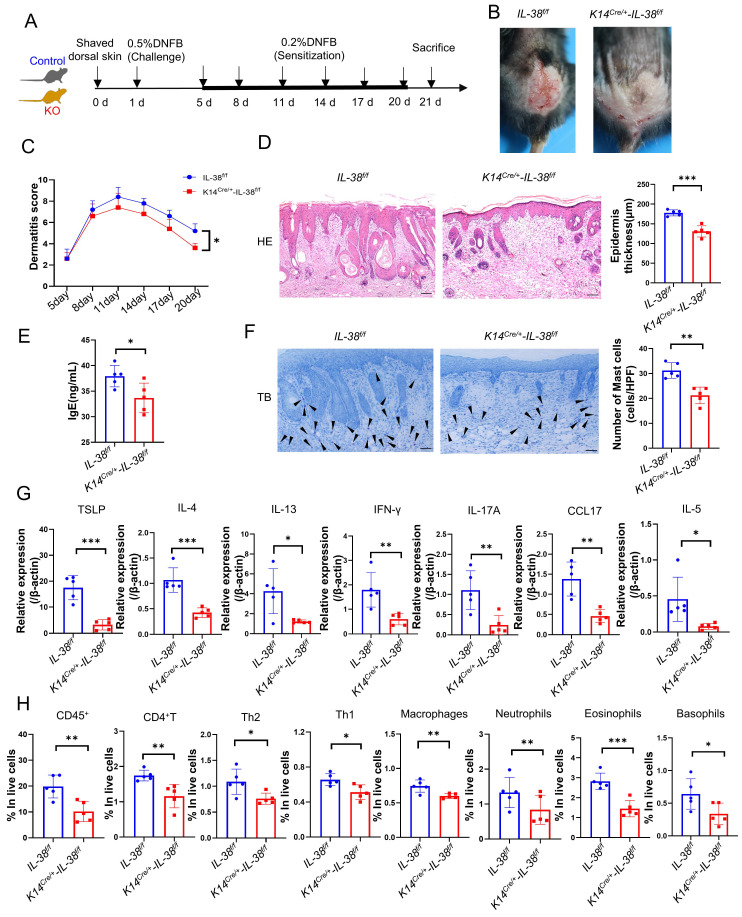
** Keratinocyte-specific knockout of IL-38 attenuated DNFB-induced AD symptoms and development by suppressing the expression of inflammatory cytokines and infiltration of immune cells in the AD lesion.** (A) Established scheme of DNFB-induced AD mode of* K14^Cre/+^-IL-38^f/f^* and* IL-38^f/f^* mouse. (B) Representative graph of dorsal skin lesions status in* K14^Cre/+^-IL-38^f/f^* and* IL-38^f/f^* mouse after DNFB-induced AD. (C) The total dermatitis scores in dorsal lesion areas of *K14^Cre/+^-IL-38^f/f^* (n=5) and* IL-38^f/f^* (n=5) mice were assessed according to the severity of the four symptoms, erythema/hemorrhage, edema, excoriation/erosion, and scaling/dryness after DNFB-induced AD. (D) H&E staining in *K14^Cre/+^-IL-38^f/f^* (n=5) and* IL-38^f/f^* (n=5) mice after DNFB-induced AD to detect epidermal proliferation in the skin lesion area. Scale bars represent 100 μm. (E) Determination of IgE in serum of *K14^Cre/+^-IL-38^f/f^* (n=5) and* IL-38^f/f^* (n=5) mice by ELISA after DNFB-induced AD. (F) Toluidine blue staining (TB) after DNFB-induced AD to measure alterations in the number of mast cells in the lesion area of *K14^Cre/+^-IL-38^f/f^* (n=5) and* IL-38^f/f^* (n=5) mice, arrows in the graph indicate mast cells. Scale bars represent 50 μm. (G) Relative expression of multifarious inflammation cytokines in* K14^Cre/+^-IL-38^f/f^* (n=5) and* IL-38^f/f^* (n=5) mice lesion was quantified by RT-qPCR after DNFB-induced AD. (H) Flow cytometry assay the proportion of infiltrating immune cells in the total number of live cells in *K14^Cre/+^-IL-38^f/f^* (n=5) and* IL-38^f/f^* (n=5) mice of lesion skin after DNFB-induced AD. Error bars represent the mean ± SD. *p < 0.05; **p < 0.01; ***p < 0.001; p values were calculated using Student's t test or two-way ANOVA.

To investigate the role of IL-38 in AD, we generated AD models in *K14^Cre/+^-IL-38^f/f^* and *IL-38^f/f^* mice by DNFB (Figure 3A-B). Dermatitis scores were assessed according to the severity of skin, starting from day 5 of modelling 34. The cumulative dermatitis score was computed and was found to be relatively higher in *IL-38^f/f^* than in *K14^Cre/+^-IL-38^f/f^* mice ((Figure 3C). Furthermore, H&E staining also demonstrated a significant epidermal thickening in *IL-38^f/f^* mice (Figure 3D). Excessive serum IgE secretion is a key characteristic of AD 36, and our ELISA results revealed elevated levels of IgE expression in the serum of *IL-38^f/f^* mice compared to *K14^Cre/+^-IL-38^f/f^* (Figure 3E). Mast cell activation is closely associated with IgE, and activated mast cells can exacerbate AD development by increasing vascular permeability, facilitating immune cell infiltration, and secreting inflammation-related cytokines 34, 37, 38. To explore this further, we stained mast cells with toluidine blue (TB) and observed a significant reduction in mast cell numbers after IL-38 knockout (Figure 3F). In AD-lesions, the production of TSLP, a critical activator of Th2 inflammation 39, as well as Th2-related cytokines such as IL-4, IL-5, and IL-13 can enhance AD progression 40. Furthermore, the production of cytokines such as CC17 41, IFN-γ 42 and IL-17 43 adds to the development of AD. We measured the expression of these cytokines in the AD-lesion by quantitative real-time PCR (Figure 3G). The results showed a significant increase in the expression of AD-related inflammatory cytokines of *IL-38^f/f^* mice compared to *K14^Cre/+^-IL-38^f/f^* mice. Moreover, abundant inflammatory cytokines further promoted infiltration of immune cells into AD-lesion such as macrophages 44, eosinophils 11, basophils 45 and neutrophils 46. We assessed immune cells infiltration in AD-lesions by flow cytometry. The results showed that the percentage of infiltrated immune cells in skin lesion of *IL-38^f/f^* mice was more than *K14^Cre/+^-IL-38^f/f^* mice (Figure 3H and Figure S3A-B). Collectively, these findings suggest that IL-38 keratinocyte-specific knockout suppressed the DNFB-induced AD-like skin inflammation. Furthermore, to test whether the progression of AD is directly mediated by IL-38, we subcutaneously injected recombinant murine IL-38 protein (rmIL-38) or vehicle into the backs of DNFB-induced mice. The results showed that subcutaneous injection of rmIL-38 promoting lesion scores and the symptoms of DNFB-induced AD, by increasing epidermal thickness, and the serum levels of IgE as well as the inflammatory infiltrate in AD-lesion (Figure S4A-G). The results indicated that IL-38 can directly influence the development of AD.

Taken together, these findings suggest that IL-38 plays a significant role in the development of AD. Moreover, keratinocyte-specific knockout of IL-38 effectively suppressed DNFB-induced AD-like skin inflammation by reducing the expression of inflammatory cytokines and infiltration of immune cells in skin lesions.

### IL-38 regulates Th2 differentiation by facilitating the migration of LCs to promote the progression of AD

**Figure 4:**
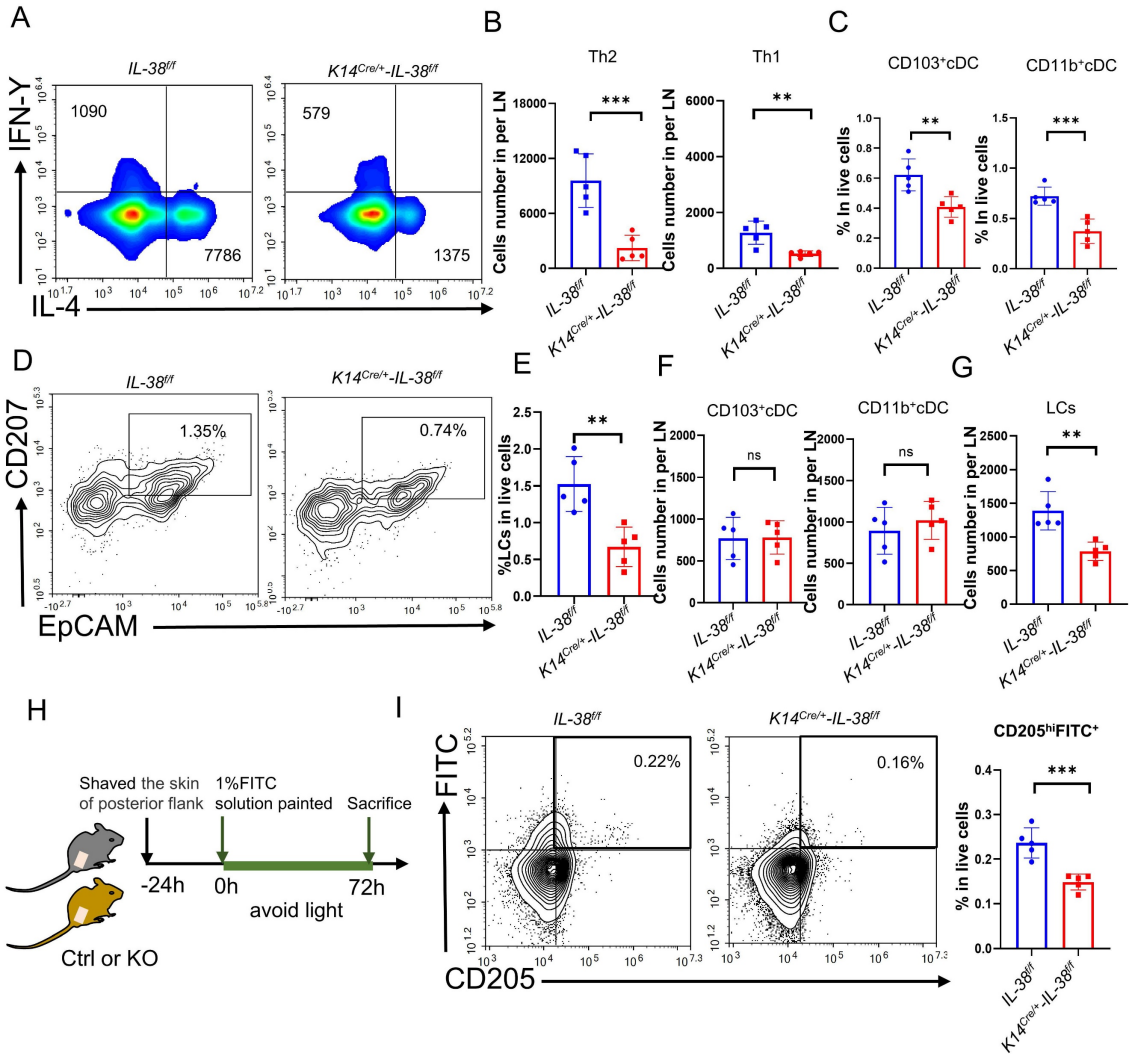
** IL-38 facilitates the migration of LCs to lymph nodes and influences CD4^+^T cells differentiation especially Th2 cells in lymph nodes.** (A) Representative graph of flow cytometry about Th1 and Th2 cells differentiation in lymph nodes of *K14^Cre/+^-IL-38^f/f^* and* IL-38^f/f^* mice after DNFB-induced AD. (B) Statistical graph of Th1 and Th2 cells differentiation in lymph nodes of *K14^Cre/+^-IL-38^f/f^* (n=5) and* IL-38^f/f^* (n=5) mice analyzed by flow cytometry after DNFB-induced AD. (C) Flow cytometry assay the proportion of CD103^+^cDC and CD11b^+^cDC cells in the total number of live cells in* K14^Cre/+^-IL-38^f/f^* (n=5) and *IL-38^f/f^* (n=5) mice of lesion skin after DNFB-induced AD. (D and E) Flow cytometry analysis the proportion of LCs in the total number of live cells on AD-lesion skin in *K14^Cre/+^-IL-38^f/f^* and* IL-38^f/f^* mice after DNFB-induced AD. (F and G) Statistical graph on number of CD103^+^cDC, CD11b^+^cDC and LCs migrate to skin-draining lymph nodes in *K14^Cre/+^-IL-38^f/f^* (n=5) and* IL-38^f/f^* (n=5) mice after DNFB-induced AD. (H) Schematic diagram of the protocol for measuring migration of LCs after FITC painting in mice. (I) *K14^Cre/+^-IL-38^f/f^* and* IL-38^f/f^* mice were treated with 1% FITC (dissolved in acetone and dibutyl phthalate), respectively. Subsequently, the number of CD205^hi^FITC^+^ migrating to lymph nodes in* K14^Cre/+^-IL-38^f/f^* (n=5) and* IL-38^f/f^* (n=5) mice was detected and analyzed by flow cytometry. Error bars represent the mean ± SD. ns, not significant; **p < 0.01; ***p < 0.001; p values were calculated using Student's t test.

AD is a cutaneous inflammatory disease characterized predominantly by Th2 immune responses, which involve the differentiation of CD4^+^T cells from lymph nodes into Th2 subsets, resulting in a disrupted Th1/Th2 balance and a subsequent cascade of immunological responses 8. To understand how IL-38 promotes AD development, we assessed the differentiation of Th1 and Th2 cells in the lymph nodes (LN) of *K14^Cre/+^-IL-38^f/f^* and *IL-38^f/f^* mice after DNFB induced (Figure 4A and Figure S3B). Our findings showed that *IL-38^f/f^* mice had a much higher number of Th1 and Th2 cells in their lymph nodes than* K14^Cre/+^-IL-38^f/f^* mice, with a considerable increase in Th2 cell differentiation, after DNFB induced (Figure 4B and Figure S5A). The activation of T cells mainly depends on skin dendritic cells (DC) transport cutaneous antigen to skin-draining lymph nodes. Therefore we analyzed the number of skin DC in the AD-lesion and migrating to skin-draining lymph nodes by flow cytometry after DNFB induced in both* K14^Cre/+^-IL-38^f/f^* and *IL-38^f/f^* mice 47-49. The results showed that the ratio of epidermal LCs and dermal DC such as CD103^+^cDC and CD11b^+^cDC in the lesion of* IL-38^f/f^* mice all more than *K14^Cre/+^-IL-38^f/f^* mice (Figure 4C-E). However, surprisingly, there was no difference in the number of CD103^+^cDc and CD11b^+^cDC migrating to lymph nodes in *IL-38^f/f^* mice compared to *K14^Cre/+^-IL-38^f/f^* mice (Figure 4F), but the number of LCs was significantly higher (Figure 4G). Moreover, IL-38 as the latest member of the IL-36 subfamily, is highly expressed in the skin, secreted by epidermal keratinocytes, and absent in the lymph nodes (Figure 1A). LCs are positioned within the epidermis with keratinocytes, these implied that IL-38 had the potential to affect the differentiation of CD4^+^T in LN due to modulated epidermal LCs migrating.

Fluorescein isothiocyanate (FITC) has been widely used as a fluorescent marker to assess LC migration *in vivo* 50 and can be captured by LCs, taking at least 24 hours to migrate to the draining lymph nodes for detection 51. To further investigate the potential role of IL-38 in promoting epidermal LCs migration, we used flow cytometry to detect LCs migrating to LN after applying 1% FITC to the lateral abdomen of *K14^Cre/+^-IL-38^f/f^* and *IL-38^f/f^* mice (Figure 4 H). CD205 is the receptor specifically expressed in dendritic cells and is highly expressed in Langerhans cells and low expressed in dermal DC 48. Thus the LCs migrating to LN can be separated by CD205^hi^FITC^+^. Remarkably, the result showed that CD205^hi^FITC^+^cell population in the lymph nodes of *IL-38^f/f^* mice were significantly higher compared to *K14^Cre/+^-IL-38^f/f^* mice (Figure 4I and Figure S5B). These observations indicate that IL-38 may regulate the movement of epidermal LCs, ultimately affecting the differentiation of Th1 and Th2 cells.

### IL-38 upregulates the expression of CCR7 to promote LCs migration

**Figure 5:**
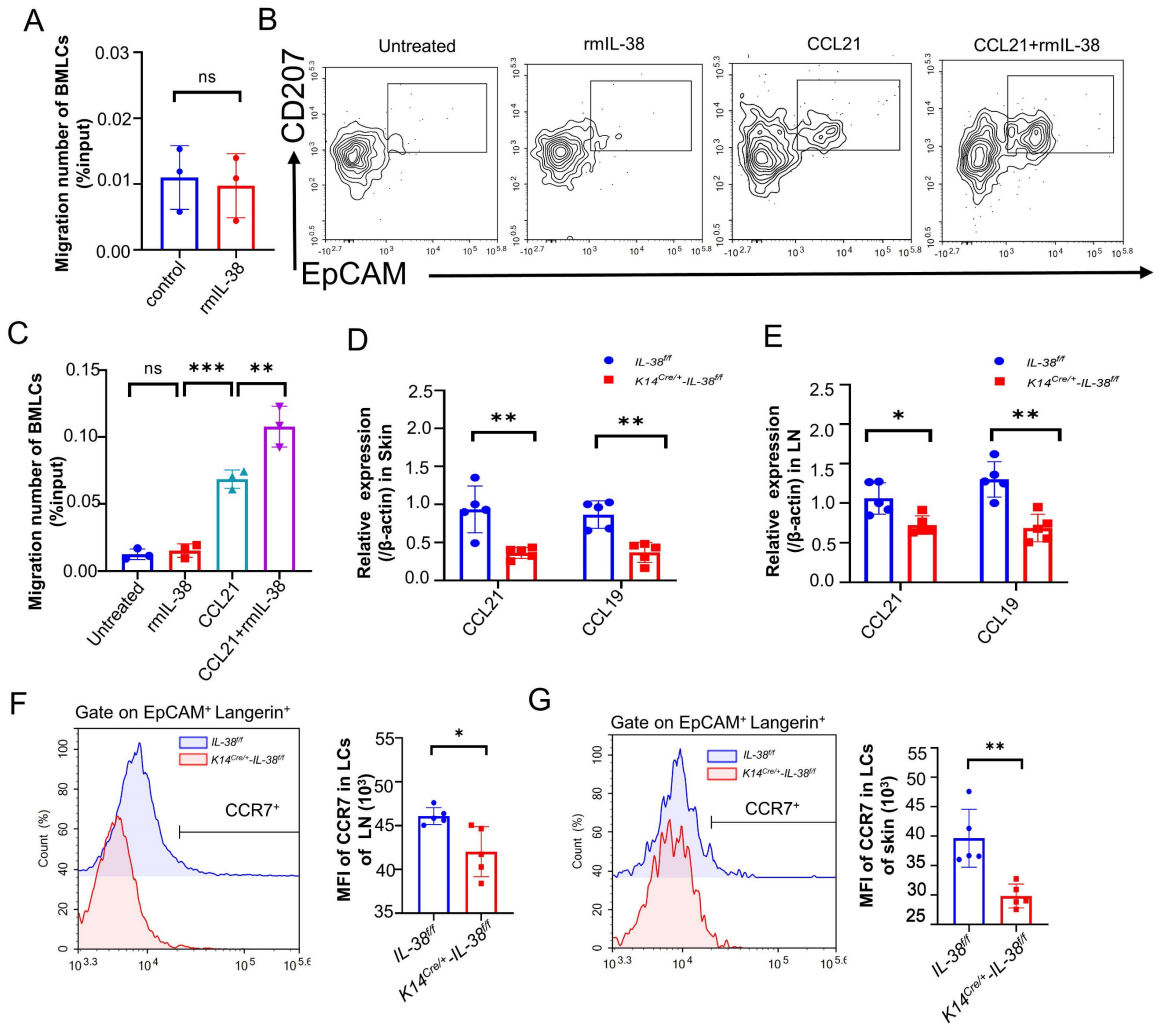
** IL-38 has non-chemotactic properties of LCs, but can upregulate CCR7 expression to promote migration of LCs, aggravating the progression of AD.** (A) Bone marrow cells were isolated *in vitro* and induced into LCs (BM-LCs). 100 ng/ml rmIL-38 or equal volume of vehicle was added to the lower chamber of the Transwell and incubated at 37°C in a 5% CO_2_ incubator for 3 h. The number of BM-LCs migrating into the lower chamber was collected and analyzed by flow cytometry (n=3). (B and C) Bone marrow cells were isolated *in vitro* and induced into LCs (BM-LCs). BM-LCs were inserted in the Transwell upper chamber with being pretreated with rmIL-38 or vehicle for 24 hours. The lower chamber of Transwell was loaded recombinant mouse CCL21 protein or vehicle. After incubation, the number of cells migrating to the bottom chamber was determined by flow cytometry (n=3). (D) Relative expression of CCR7L on AD-lesion skin in* K14^Cre/+^-IL-38^f/f^* (n=5) and* IL-38^f/f^* (n=5) mice were quantified by RT-qPCR after DNFB-induced AD. (E) Relative expression of CCR7L on lymph nodes in* K14^Cre/+^-IL-38^f/f^* (n=5) and* IL-38^f/f^* (n=5) mice were quantified by RT-qPCR after DNFB-induced AD. (F) Flow cytometry measurement of CCR7 expression in LCs migrating to lymph nodes in* K14^Cre/+^-IL-38^f/f^* (n=5) and* IL-38^f/f^* (n=5) after DNFB-induced AD. (G) Flow cytometry measurement of CCR7 expression in LCs on AD-lesion skin in* K14^Cre/+^-IL-38^f/f^* (n=5) and* IL-38^f/f^* (n=5) after DNFB-induced AD. Error bars represent the mean ± SD. ns, not significant; *p < 0.05; **p < 0.01; ***p < 0.001; p values were calculated using Student's t test or One-way ANOVA and Two-way ANOVA.

Keratinocytes play a critical role in the recruitment of LCs within the epidermis 52. We therefore wondered whether IL-38, which is secreted mainly by keratinocytes and highly expressed in the skin, is also involved in the recruitment of LCs to the lesion area, thus exacerbating the progression of AD. Referring to relevant literature 53-55, we isolated mouse bone marrow (BM) and induced its differentiation into LCs (BM-LCs) *in vitro* (Figure S5C). BM-LCs were placed in the upper chamber of a Transwell system, with recombinant mouse IL-38 protein (rmIL-38) or vehicle added to the lower chamber. Subsequently, the migrated BM-LCs from the lower chamber were collected and quantified by flow cytometry (Figure 5A). Surprisingly, the presence of rmIL-38 in the lower chamber didn't affect the number of BM-LCs migrating to the lower chamber compared to the control group without rmIL-38. This implies that IL-38 could not directly recruit LCs or controlling their direction of migration. The IL-36 subfamily members such as IL-36α, IL-36β, and IL-36γ can activate GM-CSF-induced dendritic cells through autocrine signaling 56, 57. Hence, we speculated whether IL-38 directly acts on LCs, influencing the expression of related molecules and subsequently promoting their migration. We induced BM-LCs and treated with rmIL-38 or vehicle in advance, then placed these BM-LCs in the upper chamber of the Transwell system, with chemokine CCL21 protein or vehicle in the lower chamber. After incubation, the number of BM-LCs migrating to lower chamber was detected by flow cytometry. The findings showed that, under CCL21 loaded in lower chamber, compared to vehicle pretreatment BM-LCs there was a statistically significant increase in the number of BM-LCs migrating to the lower chamber after IL-38 pretreatment (Figure 5B-C). Moreover, the number of BM-LCs migrating to lower chamber was no obvious change when IL-38 and vehicle pretreatment BM-LCs under without CCL21in lower chamber stimulation (Figure 5B-C). Taken together, these results implied that IL-38 may affect the receptor associated with the migration of LCs to promote LCs migration.

CCL21 and CCL19(also known as CCR7L), the specific ligands of C-C chemokine receptor type 7 (CCR7), control DC transporting to draining lymph nodes, initiating adaptive immunity 55. The CCR7-CCL19/21 axis has emerged as a crucial component in immune cells transport to lymph nodes, and dysregulated CCR7 expression in DC can disrupt their transportation, leading to inflammatory diseases 58. LCs are a specialized subset of DC so we speculated that IL-38 might influence the CCR7-CCL19/21 axis to affect the migration of LCs. we employed DNFB to establish AD models in *K14^Cre/+^-IL-38^f/f^* and *IL-38^f/f^* mice. RT-qPCR analysis of CCR7L expression from AD-lesions and lymph nodes. The results showed that a significant reduction in the expression of CCR7L in AD-lesion (Figure 5D) and lymph nodes (Figure 5E) upon IL-38 keratinocyte-specific knockout. These changes suggest the potential involvement of IL-38 in CCR7-CCL19/21 axis regulation. CCR7L are predominantly secreted by the high endothelial venules (HEVs) and reticular stromal cells (fibroblastic reticular cells, FRCs) in the T-cell-rich paracortical regions of LNs, as well as in the thymus and the spleen 59. Considering these aspects and the expression of CCR7 in LCs, we suspect that IL-38 affects CCR7 receptor to regulate the migration of LCs. To further verify the conjecture, we used flow cytometry to measure CCR7 expression in LCs from the lymph nodes and epidermis after DNFB-induced in *K14^Cre/+^-IL-38^f/f^* and *IL-38^f/f^* mice (Figure 5 F and G). The results showed that the CCR7 expression in LCs of the AD-lesions and lymph nodes both reduced in *K14^Cre/+^-IL-38^f/f^* mice compared to* IL-38^f/f^* mice. Collectively, these findings suggest that IL-38 upregulate the expression of CCR7 to modulate CCR7-CCL19/21 axis, which in turn promotes LCs migration and regulates Th2 cell differentiation in LN.

### IL-38 activates the IRAK4/NF-κB pathway to upregulate CCR7 to promote migration of LCs dependent on IL-36R

**Figure 6:**
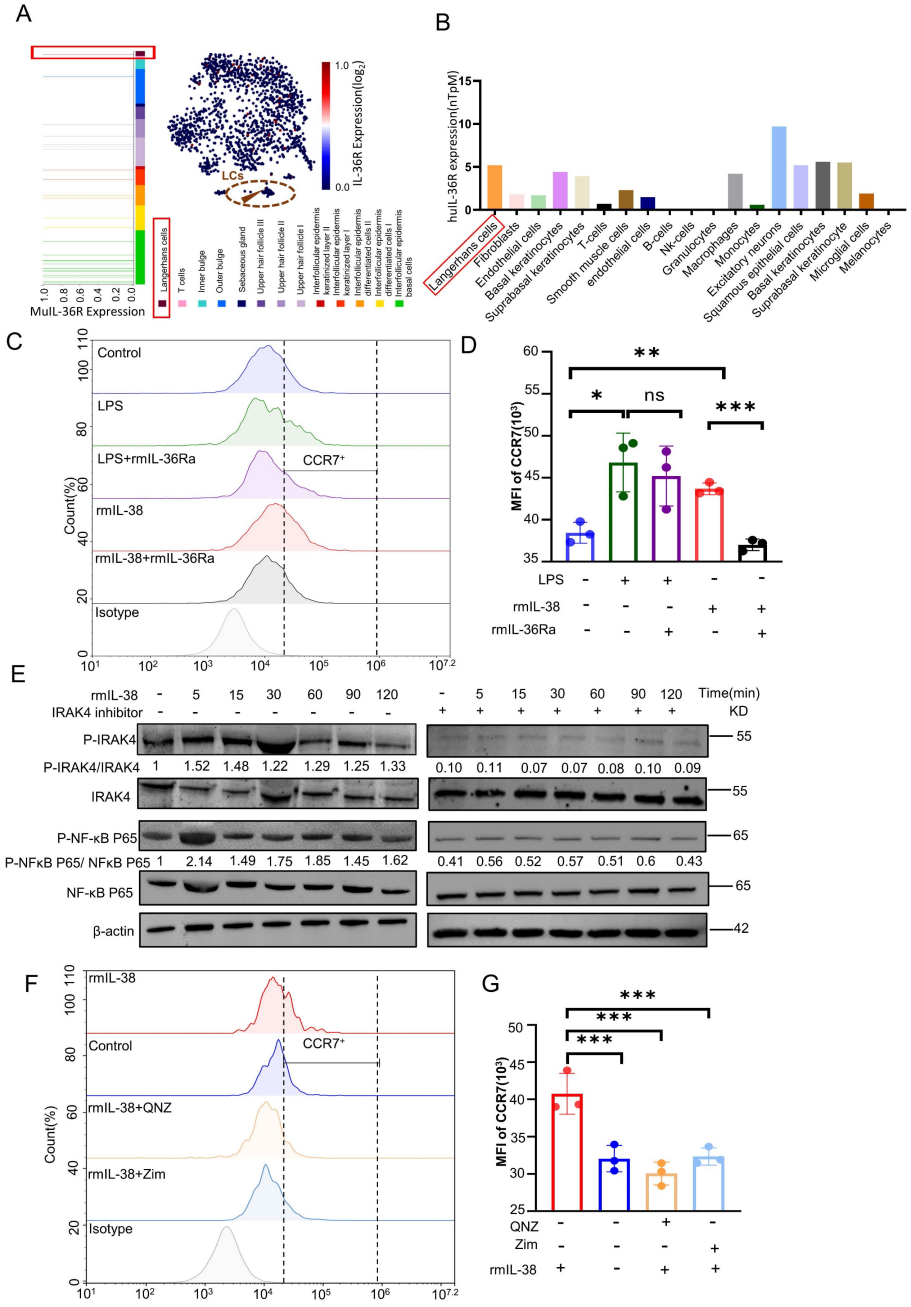
** IL-38 depends on IL-36R, triggers IRAK4/NF-κB phosphorylation, and promotes LCs migration by elevating the expression of CCR7.** (A) Retrieval of open data from Linnarsson Lab mouse epidermal single cell sequencing and analysis of IL-36R expression on mouse LCs. (B) Search and analysis of the HPA database for IL-36R expression in different human cells. (C and D) After isolation of mouse bone marrow cells induced into BM-LCs, rmIL-38, rmIL-36Ra, LPS or vehicle was added to stimulate for 24h. The expression of CCR7 in BM-LCs was detected and analyzed by flow cytometry (n=3). (E) Mouse bone marrow primary cells were isolated and induced as BM-LCs. Then, stimulated BM-LCs by rmIL-38 or vehicle, under IRAK4 inhibitor Zimlovisertib or vehicle pretreatment for 2h. Proteins were extracted in different time points and the expression of IRAK4/NF-κB phosphorylation were assayed by WB. The normalized intensities of the phosphorylated IRAK4 and NF-κB p65 relative to their total forms are presented. (F and G) Following induction into BM-LCs *in vitro*, rmIL-38 or vehicle was added in BM-LCs to stimulate for 24h, under IRAK4 inhibitor Zimlovisertib, NF-κB inhibitor QNZ or vehicle pretreatment for 2h. After that, the expression of CCR7 in BM-LCs was detected and analyzed by flow cytometry (n=3). (Zim, Zimlovisertib). Error bars represent the mean ± SD. ns, not significant; *p < 0.05; **p < 0.01; ***p < 0.001; p values were calculated using One-way ANOVA.

We previously identified the IL-38 could bind to IL-36R and induced skin inflammation in response to external stimuli 22. To further explore the mechanism of IL-38 upregulation of CCR7, we analysis of the HPA database and Linnarsson Lab mouse epidermal single-cell sequencing. We found IL-36R, the receptor of IL-38, was expressed in both mouse and human LCs (Figure 6A and B). Thus, we speculated IL-38 upregulated the expression of CCR7 in LCs dependent on IL-36R. To investigate this, we induced BM-LCs and treated with rmIL-38 or LPS (as a positive control 60, 61) under added IL-36 receptor antagonist IL-36Ra protein (rmIL-36Ra) or vehicle 62, 63. Then the expression of CCR7 in BM-LCs was detected by flow cytometry. The results showed that CCR7 expression on BM-LCs was significantly increased in both the LPS-positive control group and rmIL-38 group compared to the control group. However, CCR7 expression in the rmIL-38 group was significantly reduced upon the addition of rmIL-36Ra, while there was no significant change in the LPS group (Figure 6 C-D). These findings indicate that IL-38 upregulates CCR7 in an IL-36R-dependent manner.

IRAK4 plays a crucial role in Toll-like receptor (TIR) signaling, which is also recognized as a common NF-κB activator in innate and adaptive immunity 59, 64, 65. NF-κB is another key player in inflammatory diseases and can subunit P65 translocates to the nucleus upon stimulation, inducing CCR7 transcription through its binding site in the CCR7 promoter 59, 66. IL-1 receptor family member (ILR), IL-36R, contains an extracellular immunoglobulin (Ig) domain and an intracellular TIR domain 17. Upon ligand binding, ILR dimerize through their TIR domains, inducing the recruitment of the TIR domain-containing adaptor protein, which couples to downstream protein kinases such as IRAK4, triggering downstream signaling, including NF-κB activation 67-69.

Thus, we hypothesized that IL-38 depend onIL-36R upregulated CCR7 in LCs via the IRAK4/NF-κB pathway. To test this hypothesis, we induced the BM-LCs *in vitro* and treated with rmIL-38. Then, western blot to analyze the expression of IRAK4, NF-κB, phosphorylated IRAK4 and NF-κB in BM-LCs (Figure 6 E). The results showed that the expression of phosphorylated IRAK4 and NF-κB in BM-LCs was increased after rmIL-38 stimulation. However, pretreating with IRAK4 inhibitor Zimlovisertib or NF-κB inhibitor QNZ, the phosphorylated expression of IRAK4 and NF-κB significantly decreased (Figure 6 E and Figure S5D). Moreover, the IRAK4 inhibitor Zimlovisertib dampened IL-38-induced phosphorylation of NF-κB in BM-LCs (Figure 6 E). These findings provide evidence that IL-38 activates the IRAK4/NF-κB signaling pathway.

To investigate the dependence of IL-38-induced CCR7 upregulation by IRAK4/NF-κB signaling pathway, we induced BM-LCs *in vitro* and pretreated with Zimlovisertib, QNZ or vehicle. After that, we treated with rmIL-38 or vehicle for 24 hours, and the expression of CCR7 on BM-LCs was evaluated by flow cytometry (Figure 6F-G). Our results demonstrated a significant upregulation of CCR7 expression on BM-LCs upon rmIL-38 treatment compared to the control group. However, no significant difference in CCR7 expression was observed between the rmIL-38 group and the control group when the Zimlovisertib or QNZ was added. These data indicated that IL-38 activated the IRAK4/ NF-κB signaling pathway to upregulate CCR7 in LCs dependent on IL-36R.

### Blockage of the IRAK4 signaling attenuated the DNFB-induced the progression of AD

**Figure 7:**
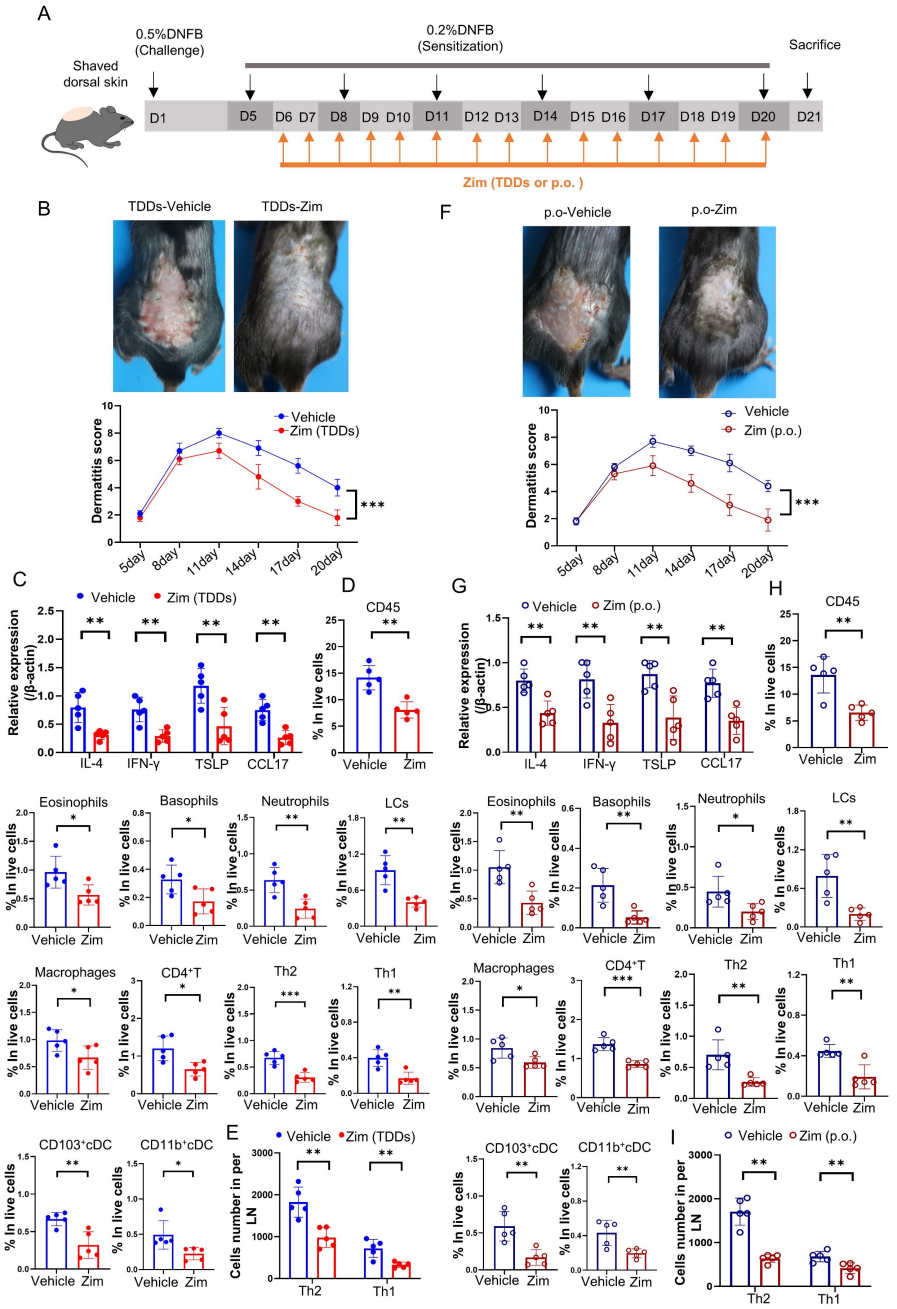
** Application of IRAK4 inhibitor attenuates DNFB-induced AD symptoms.** (A) The schematic of the scheduled treatment in DNFB-induced AD mode of wild-type mice by application IRAK4 inhibitor Zimlovisertib (Zim, Zimlovisertib). (B) Mice were treated with IRAK4 inhibitor Zimlovisertib or vehicle by TDDs in AD-lesion after DNFB-induced AD. The upper graph representative of dorsal skin lesions status, while the dermatitis scores in dorsal lesion areas in the lower (n=5) (TDDs, Transdermal drug delivery systems). (C) Relative expression of multifarious inflammation cytokines on AD-lesion skin in mice which were treated by IRAK4 inhibitor Zimlovisertib or vehicle of TDDs in AD-lesion after DNFB-induced AD were quantified by RT-qPCR (n=5). (D) Flow cytometry assay number of infiltrating immune cells in mice of lesion skin which were treated with IRAK4 inhibitor Zimlovisertib or vehicle by TDDs in AD-lesion after DNFB-induced AD (n=5). (E) Flow cytometry assay number of Th1 and Th2 cells differentiation in lymph nodes of mice treated with IRAK4 inhibitor Zimlovisertib or vehicle by TDDs in AD-lesion after DNFB-induced AD (n=5). (F) IRAK4 inhibitor Zimlovisertib or vehicle were given orally (p.o.) to mice after DNFB-induced AD. The upper graph representative of dorsal skin lesions status and the dermatitis scores of AD-lesion skin in the lower (n=5). (G) Relative expression of multifarious inflammation cytokines on AD-lesion skin in mice treated with IRAK4 inhibitor Zimlovisertib (p.o.) or vehicle (p.o.) after DNFB-induced AD. (n=5). (H) Flow cytometry assay number of infiltrating immune cells of lesion skin after treated by IRAK4 inhibitor Zimlovisertib (p.o.) or vehicle (p.o.) in DNFB-induced mice. (n=5). (I) Flow cytometry assay number of Th1 and Th2 cells differentiation in lymph nodes after treated by IRAK4 inhibitor Zimlovisertib (p.o.) or vehicle (p.o.) in DNFB-induced mice. (n=5). Error bars represent the mean ± SD. *p < 0.05; **p < 0.01; ***p < 0.001; p values were calculated using Student's t test or One-way ANOVA and Two-way ANOVA.

To further elucidate the role of the IRAK4/NF-κB axis in AD, we combined the IRAK4 inhibitor Zimlovisertib and the NF-κB inhibitor QNZ to treat DNFB-induced AD mice by either transdermal drug delivery systems (TDDs) or oral administration (p.o.). The results found AD-like skin inflammation were alleviated after treating with Zimlovisertib and QNZ in both TDDs and p.o. (Figure S6A-I). Increasing evidence suggests that targeting IRAK4 is an attractive therapeutic concept for several immune-mediated inflammatory diseases such as AD 70. IRAK4 is regarded as NF-κB activator and inhibition of IRAK4 activity with IRAK4 inhibitor represents a prospective and safe therapeutic strategy for inflammatory disorders 71. We treated DNFB-induced AD mice only with IRAK4 inhibitor Zimlovisertib also by either TDDs or oral administration (Figure 7 A). The results showed that the application of Zimlovisertib in TDDs to the AD-like lesion area led to a decrease in the dermatitis score (Figure 7 B). Additionally, the expression of inflammatory cytokines (Figure 7 C) and percentage of infiltration of immune cells (Figure 7 D) significantly decreased in the AD-lesion. Furthermore, the differentiation of Th2 and Th1 in the lymph nodes was also reduced (Figure 7 E). Similarly, oral administration of Zimlovisertib, which has the same effect, relieves the symptoms of AD (Figure 7 F-I). Overall our results demonstrate that blockage of the IRAK4 signaling can suppress the DNFB-induced AD-like skin inflammation both in TDDs and p.o., implying their potential and clinical significance.

## Discussion

AD also known as atopic eczema, with a complex etiology, characterized by symptoms starting before the age of 6 in approximately 80% of patients and lasting for many years, with a lifetime prevalence of up to 20% 72-74. Notably, AD is often associated various non-atopic comorbidities, including psychiatric disorders, which significantly impact patients' daily lives 75, 76. Although the last decade has seen a surge in clinical trials examining novel therapies for the treatment of moderate-to-severe AD, unfortunately there is no cure for atopic dermatitis 77. However, medications that regulate inflammation and immune system activity can improve or resolve symptoms that are not well controlled with the non-medication treatments 78.

The IL-1 family plays a crucial role in both innate and adaptive immunity and is involved in the regulation of the pathogenesis of several skin diseases. Literature has reported that IL-33, a cytokine in the IL-1 family same as IL-38, can activate DC, enhances their migration to draining lymph nodes, and induces Th2 cell differentiation 79. It is reasonable to assume that IL-38, a new member of the IL-1 family and highly expressed in the skin, may contribute to the regulation of skin disease development. However, the specific involvement of IL-38 in AD is unexplored. Therefore, our study aims to fill this research gap. Through this research, we present the first evidence demonstrating the role of IL-38 in the development of AD. Moreover, we elucidate its mechanism in promoting LCs migration, thereby facilitating AD progression.

We successfully generated *K14^Cre/+^-IL-38^f/f^* and *IL-38^f/f^* mice to specifically knockout IL-38 in the keratinocyte by Cre-loxp system. Keratinocytes are vital in maintaining the epidermal barrier, and alterations in gene expression within these cells can disrupt skin development and lead to inflammatory diseases in mice. Interestingly, *K14^Cre/+^-IL-38^f/f^* mice did not exhibit any abnormalities in the skin barrier or spontaneous cutaneous inflammation. This suggests that deleting IL-38 in the keratinocyte has minimal impact on barrier function and immune responses in normal conditions. However, as previously reported, patients carrying a 175-kb deletion on chromosome 2q, encompassing the genes coding for IL-36γ, IL-36α, IL-36β, IL-36Ra, IL-38, and IL-1Ra, suffer from severe autoinflammatory syndrome 80. Hence, we propose that under normal conditions, members of the IL-1 family may possess mechanisms that maintain inflammatory cytokines balance and ensure skin homeostasis. However, when the skin is exposed to external stimuli that compromise the barrier and disrupt inflammatory cytokines homeostasis, inflammation may occur. Thus, the potential mechanism of IL-1 family in skin remains explored.

Although most studies demonstrate that IL-38 has anti-inflammatory properties by, for example, blocking IL-1beta maturation, the biological functions of IL-38 are more complex than only inhibiting the inflammatory response, and some *in vivo* studies demonstrate that this cytokine favors disease progression 81. The biological function of IL-38 is still controversial, and there is no definitive conclusion as to whether it is an agonist or antagonist and its signal pathway 82, 83. Exploring published literature on IL-38, we discovered its high expression in inflammatory bowel disease tissues compared to normal tissues, where it inhibits intestinal inflammation 84. Additionally, IL-38 is lowly expressed in squamous cell carcinomas, and keratinocyte-specific knockout of IL-38 inhibits tumor cell proliferation, migration, and the expression of inflammatory cytokines, as well as immune cell infiltration 22. Blockade of IL-38 activity can re-activate immunostimulatory mechanisms in the tumor microenvironment leading to immune infiltration, the generation of tumor-specific memory and abrogation of tumor growth 85. Moreover, IL-38 ablation ameliorates autoimmune encephalomyelitis and reduces inflammation cytokines 28. Even IL-38 can act as a growth factor to regulate intestinal stem cell homeostasis 81. Here, our research findings reveal that IL-38 was downregulated in AD lesion but can bind to IL-36R and activate the IRAK4/NF-κB signaling pathway to upregulate CCR7 in LCs, promoting the migration of epidermal LCs to draining lymph nodes after DNFB induction, further facilitating the development of AD. Thus, considering these, decreased local levels of IL-38 in inflammatory conditions may not definitely seem at odds with the proposed promoting inflammatory conditions of this cytokine, and further investigation is required to elucidate the precise mechanism.

During AD development, allergen exposure triggers cytokine release from keratinocytes, activating Th2 cells and resulting in the production of Th2-related cytokines, IgE synthesis, mast cell degranulation, immune cell infiltration into lesions, collectively driving inflammatory cytokine production, compromising the skin barrier, and promoting AD 86-89. Furthermore, IL-4 and IL-13 directly stimulate sensory neurons, causing itching. Scratching induces keratinocytes to produce additional inflammatory cytokines, such as CCL17 and TSLP, exacerbating AD 89. Given this, we measured the infiltration of inflammatory cytokines, immune cells, and serum IgE levels in *K14^Cre/+^-IL-38^f/f^* and *IL-38^f/f^* mice after DNFB induced. The results demonstrated reduced levels of inflammatory cytokines and infiltration of immune cells in the AD-lesions after IL-38 keratinocyte-specific knockout, along with decrease in serum IgE levels.

The progress of AD primarily involves the capture of allergens by LCs, which then move to the lymph nodes and trigger the differentiation of CD4^+^T cells, leading to inflammation. Our study showed that *IL-38^f/f^* mice had more LCs in the AD-lesions compared to *K14^Cre/+^-IL-38^f/f^* mice. This may be associated with increased levels of systemic inflammation. In inflammatory conditions, increasing amounts of inflammatory cytokines accumulate in the skin lesion, further promoting infiltration of immune cells into AD lesions. Based on our findings, it is suggested that the application of IL-36R receptor antagonist, IRAK4 or NF-κB inhibitor to the AD lesion could potentially alleviate the symptoms following DNFB treatment. Since IL-36R receptor antagonist (IL-36Ra) is susceptible to inactivation and recombinant proteins have relatively short half-lives, we opted for an approach of IRAK4 or NF-κB inhibitor administered via TDDs and oral routes in DNFB-induced AD. Both modes of administration demonstrated a reduction in DNFB-induced AD symptoms by IRAK4 inhibitor or combination with NF-κB inhibitor. NF-κB, a major inflammatory transcription factor that regulates immune response genes 90, is also involved in CCR7 transcription and can be regulated by IRAK4. IRAK4 is a member of the IL-1R-related kinase family and important for signaling in response to Toll-like receptors 91. IRAK4 is overexpressed relative to healthy people and can activate downstream molecules involved in cytokine as well as inflammatory responses, it now considered a promising target for the treatment of inflammatory diseases 92, 93. The primary objective of AD treatment is to attain and sustain a state of minimal or mild clinical symptoms that do not hinder the patient's daily activities and necessitate minimal use of medication 94. Moreover, treating with TDDs or p.o. to inhibit IRAK4 can potentially provide the suppression of inflammatory responses while maintaining adequate levels of protection against microbial infections 95. However, there are several adverse effects of currently available inhibitors of NF-κB 80. Considering this, the potential utilization of IRAK4 inhibitor in AD treatment may more promising, specifically in the development of formulations for clinical use. Our future research will primarily focus on this aspect.

It is worth mentioning that, due to experimental restrictions, we were unable to generate langerin-diphtheria toxin receptor (DTR) mice for the selective and transient depletion of LCs, thus hindering our ability to inversely validate the IL-38 facilitation of DNFB-induced AD progression whether specifically required for LCs. Additionally, although we observed that subcutaneous injection of rmIL-38 can promote DNFB-induced AD symptoms, due to our current experiment's designs and laboratory limitations, we did not construct IL-38 overexpressing transgenic mice to investigate whether overexpression of IL-38 in skin can spontaneously induce AD-like symptoms and corresponding verification. Exploring whether IL-38 overexpression can induce conditions like type 2 inflammation will be also a critical focus of our future experiments. Moreover, the signaling pathway we describe was specifically identified in DNFB-induced AD-like skin inflammation. Therefore, it is important to consider that other inflammatory stimuli may elicit distinct immune reactions and involve different cell populations with unique ligand-receptor pairs. Lastly, due to laboratory constraints, we have not formulated IRAK4 inhibitors into emulsions or other dosage forms for clinical trials.

In summary, we established IL-38 keratinocyte-specific knockout mice and employed the DNFB-induced model to examine the potential impact of IL-38 on AD progression. Meanwhile, through cellular and molecular biology approaches, we uncovered the mechanisms of IL-38 in AD. Our results confirm that IL-38 activates the IRAK4/NF-κB pathway and depends on IL-36R to upregulate CCR7. This promotes LCs migration to lymph nodes and triggers CD4^+^T differentiation into Th1 and Th2, thereby generating inflammatory responses and thus promoting the development of AD. Meanwhile, IRAK4 inhibitors treated in DNFB-induced AD mice was found to alleviate AD-like symptoms, indicating a potential clinical application of these inhibitors. Our study highlights the significance of IL-38 and IRAK4 in the pathogenesis of AD. Furthermore, it offers new insights and potential targets for understanding the underlying mechanisms of AD and developing effective drugs for its clinical treatment.

## Materials and Methods

### Animals

As previously reported 22, we commissioned Cyagen Biosciences Inc. for the construction of *IL-38-floxed* (C57BL/6J-Il1f10^em1cyagen^) mice on the C57BL/6 background. *Krt14-Cre* mice (*STOCK Tg[Krt14-cre] 1Amc/J*) mice were purchased from Nanjing Biomedical Research Institute of Nanjing University (J004782). C57BL/6 mice (Wild-type, WT) were purchased from Vital River Laboratory Animal Technology Co., Ltd. (219). The animals were kept under the following controlled conditions: 12 hours of light/12 hours of darkness, temperature stabilized at 25±1°C, and free access to water and food. The experiments were carried out following the National Institutes of Health's ethical guidelines for the care and use of laboratory animals and the International Association for the Study of Pain (IASP). We made every effort to reduce the number of animals and minimize their suffering. Mouse genomic DNA was extracted from tail biopsies (Bimake, B40015), genotyping was performed by PCR assay, Gene specific primers and corresponding genotypes are listed in supplemental Table. All experimental procedures were performed following the guidelines of experimental animals from Sichuan University.

### DNFB-induced AD-like skin inflammation model and dermatitis scores

AD was induced in mice by treatment with DNFB as described previously 33, 36, 96. We induced AD-like skin inflammation in mice with minor modification. Briefly, shaving the fur of dorsal skin in mice before the experiment, AD was induced by sensitization with topical application of 100 μl of 0.5% DNFB in acetone/olive oil (4:1, v/v) to the shaved back skin on days 1, and challenge with 0.2% DNFB on days 5, 8, 11,14,17 and 20. On day 21 the mice were killed for analysis. In some experiments, mice were subcutaneously injected with 1 mg/kg of recombinant murine IL-38 (rmIL-38, Adipogen, AG-40B-0101-C010) versus control buffer into the shaved dorsal skin, commencing from day 5 and every two days for a total of consecutive 15 days. Based on criteria reported in the literature 34, the severity of the lesions on the posterior backs of the mice was assessed on the basis of four symptoms: erythema/hemorrhage, edema, excoriation/erosion, and scaling/dryness, and each symptom was scored from 0 to 3 depending on the severity of the lesion, with 0 being asymptomatic, 1 being mild (the sign is present but needs careful identification to be seen), 2 being moderate (the sign is immediately visible), and 3 for severe (the sign is very obvious). At the end of the scoring, the data were tallied. The skin clinical score was the sum of the individual scores, ranging from 0 to 12, the higher the score, the more severe the lesions.

### Skin barrier function assay in mice

The rate of transepidermal water loss (TEWL) from the skin of newborn mice was measured using the GPSkin evaporimeter (GPOWER, South Korea), according to the instructions. For toluidine blue staining, newborn mice were sacrificed and dehydrated by sequential incubation in 25%, 50%, 75%, and 100% methanol. After rehydration in PBS, they were incubated for 10 min in 0.1% toluidine blue and taken photos after detaining with PBS.

### Tissue staining and image analysis

The mouse dorsal skin samples were fixed in 4% paraformaldehyde in PBS, embedded in paraffin, sectioned, and stained with Hematoxylin and Eosin (H&E) or toluidine blue. For histopathologic examination, images were captured using an Olympus BX600 microscope (Olympus Corporation, Tokyo, Japan) and SPOT Flex camera (Olympus Corporation, Tokyo, Japan) and were analyzed with ImagePro Plus (version 6.0, Media Cybernetics) software. The epithelial thickness and infiltrating mast cells were evaluated in independent regions. For the measurement, 5 visual fields, and 5-10 measuring points were selected for each back film, and the average value was taken.

### Immunohistochemistry

The mouse dorsal skin samples were fixed in 4% paraformaldehyde in PBS. Then, the fixed sections were incubated in 3% H_2_O_2_ solution and protected from light for 10-15 min. Afterward, the sections were incubated with 5% normal goat serum at room temperature for 30 min to block non-specific antibody binding. Subsequently, the sections were stained with IL-38 (Abcam, ab180898; 1:200 dilution). The slides were then rinsed, incubated with a biotin-conjugated secondary antibody for 30 min, and incubated with horseradish peroxidase streptavidin (HRP Streptavidin) for 30 min (ZSGB-BIO). The sections were developed using a 3,3ʹ-diaminobenzidine (DAB) substrate kit (ZSGB-BIO, ZLI-9017), and hematoxylin was used for reverse staining. Images were captured using an Olympus BX600 microscope and SPOT Flex camera. ImagePro Plus was used to further quantify the DAB intensity.

### Quantitative Real-Time PCR

Total RNA of mouse skin and lymph nodes was extracted by TRIzol (Thermo Fisher Scientific) according to the manufacturer's instruction, followed by quality control using capillary electrophoresis (NanoDrop 2000; Thermo Fisher Scientific). RNA was reversed transcribed using the PrimeScript RT reagent kit with gDNA Eraser (Takara Bio; RR047A). qPCR reactions were carried out with gene-specific primers (Qing Ke Bio, primer sequences are listed in supplementary Table) mixed with TB Green™ Premix Ex Taq™ II (Takara Bio; RR820), according to the manufacturer's protocol. Samples were run in triplicates in a LightCycler96 PCR system (Roche). mRNA expression was normalized using β-actin as the reference. Analysis was performed according to the 2^-ΔΔCt^ method.

### Western blotting

Simply, the samples derived from cells and skin tissues were lysed, separated by SDS-PAGE gels (Beyotime Institute of Biotechnology), and then transferred to polyvinylidene fluoride (PVDF) membranes (Merck Millipore). For western blotting analysis, the proteins were incubated overnight with the following primary antibodies: β-actin (CST, 4970s; 1:1,000 dilution), mouse IL-38 (R&D Systems, MAB7774; 2 µg/ml), IRAK4 (CST, 4363s; 1:1,000 dilution), phospho-IRAK4 (CST, 11927s; 1:1000 dilution), NF-κB p65 (CST, 8242s 1:1,000 dilution), phospho-NF-κB p65 (CST, 3033s; 1:1000 dilution). Antibodies against phosphorylated epitopes were removed with Stripping Buffer (P0025, beyotime) before incubation with antibodies detecting the total protein. After that, the membranes were incubated by the secondary antibody which was labeled using goat anti-rabbit antibody conjugated to horseradish peroxidase (HRP) (Invitrogen, A27036 and ZSGB-BIO; 1:10,000 dilution) and further detected using ECL reagents (Merck Millipore, WBKLS0500). Band intensities in the image were quantified using ImageJ (National Institutes of Health), which included only the band intensity in the linear range.

### Flow cytometry

To obtain single-cell suspensions from dorsal skin, 2 × 3 cm sections of skin samples were incubated in 5 ml RPMI medium (Gibco) containing 500 μg/ml Liberase (Roche) for approximately 1.75 h at 37°C, chopped with sharp scissors, and incubated for an additional 15 min with 0.1 mg/ml DNase (Roche). A single-cell suspension of dorsal skin was obtained by mechanical dissociation with a gentleMACS dissociator (Miltenyi Biotech, Bergisch Gladbach, Germany), followed by filtration through 40 μm and 70 μm cell strainers. Cells were then washed once with PBS.

To obtain single-cell suspension from lymph nodes, samples were ground in 40 μm cell strainers (BD Bioscience, 352340) with 5 mL PBS solution, and then filtered with 70 μm cell strainers (BD Bioscience, 352350). Cells were washed once with PBS.

Flow cytometry was performed using the NovoCyte flow cytometer and ACEA NovoExpress™ software (ACEA Biosciences, San Diego, CA, USA) and BD LSRFortessaTM and Flow Jo™ software (BD Biosciences, USA).The single-cell suspensions were stained with the following antibodies: CD45-BV510 (1033137), CD11b-APC (17-0112-82), CD11c-APC/Cyanine7 (117324), MHCII-FITC (11-5322-81), CD64-PE/Cyanine7 (139314), Gr-1-PE/Cyanine7 (108416), CD207-PE (144204), CCR7-APC (120108), F4/80-FITC (123108), EPCAM-PE/Cyanine7 (25-5791-80), Siglec-F-FITC (155504), CD49bPE/Dazzle^TM^594 (108923), FcεRIα-PE (134308), CD3-APC/Cyanine7 (100222), CD4-PerCP/Cy5.5 (100434), CD8-PE/Cyanine7 (100722), IL-4-APC (562045), IFN-γ-FITC (505806), 7-AAD (420404). Antibodies were purchased from eBioscience and BioLegend and used at 1:100 dilution.

### Enzyme-linked immunosorbent assay (ELISA)

QuantiCyto® Mouse IgE ELISA Kit (NeoBioscience, EMC117.96) was used to detect the levels of serum IgE in mice, according to the instructions of manufacturer.

### FITC painting and *in vivo* LCs migration assay

For FITC-induced Langerhans cell migration, mice were shaved before the experiment. FITC (Sigma-Aldrich) was dissolved in a solution of acetone:dibutylphthalate (1:1, v/v; Sigma-Aldrich) to a concentration of 1% (w/v). Mice were painted on the posterior flank, lateral to the spine and over the spleen with 12μl of a 1% FITC solution for 72h and the number of migrated LCs into draining lymph nodes was enumerated by flow cytometry, as previously described 51.

### Induction of BM-derived LC-like cells (BM-LCs) and stimulation assays *in vitro*

Isolated bone marrow (BM) cells were cultured in complete RPMI-1640 medium supplemented with 10% heat inactivated fetal calf serum (Gibco), 2 mM L-Glutamine (Gibco), 50 µM 2-mercaptoethanol (Sigma-Aldrich), and 100 U/ml penicillin/streptomycin (Gibco). 5×10^5^ cells/ml were stimulated with 20 ng/ml recombinant murine GM-CSF (Peprotech) and 10 ng/ml recombinant human TGFβ1 (Peprotech) for 7 d, as previously described 53-55. BM-LCs cells were collected and spread evenly in six-well plates with or without 100 ng/mL LPS (Sigma-Aldrich), 100 ng/mL recombinant murine IL-38 (rmIL-38, Adipogen, AG-40B-0101-C010), 100 ng/mL recombinant murine IL-36Ra (rmIL-36Ra, Peprotech, 210-36RA), 10 μM QNZ (EVP4593, Selleck, S4902), 100 nM Zimlovisertib (PF-06650833, Selleck, S8531), depending on the purpose of the experiment, and the cells were collected, followed by flow cytometry or WB assay to detect the expression of the relevant molecular.

### Inhibitors treatment assay in AD

Wild-type mice were induced in AD-like skin inflammation by DNFB, and from day 6, for the transdermal drug delivery systems, 150μL 20mg/kg of Zimlovisertib (PF-06650833, Selleck, S8531), 2mg/kg of QNZ (EVP4593, Selleck, S4902) or equivalent solvents were applied within the area of the skin lesions of the mice. While for the oral regimen, mice were treated with 100μ 10mg/kg of Zimlovisertib, 1mg/kg of QNZ or equivalent solvent. The skin lesions were observed and scored until the end of modelling.

### Transwell migration assay

The migration assay was performed using 24-well Transwell plates containing 8-μm-pore size polycarbonate filters (Corning, Life Science). The cell concentration of BM-LCs was adjusted with 1640 medium and 10^6^ BM-LCs cells were added to the upper chambers and 600 μL of 1640 medium to the lower chambers. Depending on the purpose of the experiment, 100ng/mL of recombinant murine CCL21 (Peprotech, 250-13), rmIL-38 (Adipogen, AG-40B-0101-C010) or vehicle was added to the lower chamber, which was then incubated at 37°C in a 5% CO2 incubator for 3h. Cells from the lower chamber were subsequently collected. Flow cytometry was performed to detect the number of cells migrating into the lower chamber of BM-LCs. The number of spontaneously migrated LCs in the absence of chemokine was subtracted as background 62, 97.

### Statistical analysis

All statistical analyses were performed using GraphPad Prism 8.0 software. Differences between the two groups were compared using the Student's t-test. For comparisons among multiple groups, one-way ANOVA or two-way ANOVA tests were used to compare the difference. All the experimental data were expressed as mean ± standard error. * Indicates P < 0.05 for a statistically significant difference, ** indicates P < 0.01 for a statistically very significant difference, *** indicates P < 0.001 for an extremely statistically significant difference, and **** P < 0.0001 for the most statistically significant difference. ns indicates no statistical difference.

## Supplementary Material

Supplementary figures and table.

## Abbreviations

AD: atopic dermatitis
DNFB: 2,4-Dinitrofluorobenzene
DALYs: disability-adjusted life-years
CCR7: C-C chemokine receptor type 7
IgE: Immunoglobulin E
TEWL: Trans Epidermal Water Loss
TB: Toluidine blue
DC: dendritic cells
IL-38: interleukin-38
rmIL-38: recombinant murine IL-38 protein
IL-36R: interleukin-36 receptor
IL-36RA: IL-36 receptor antagonist
LCs: Langerhans cells
FITC: Fluorescein isothiocyanate
BM: bone marrow
LN: lymph nodes
TIR: Toll-like receptor
NF-κB: Nuclear factor kappa-B
IRAK4: IL-1 receptor-associated kinase 4
HEVs: high endothelial venules
FRCs: fibroblastic reticular cells
TDDs: drug delivery systems
DTR: langerin-diphtheria toxin receptor
WT: wild-type
HPA: the Human Protein Atlas database
GEO: Gene Expression Omnibus Dataset
