# Supplementary material for: IL-38 Aggravates Atopic Dermatitis via Facilitating Migration of Langerhans cells
Source: Int J Biol Sci. 2024 May 27;20(8):3094–112. doi: 10.7150/ijbs.93843 (PMC11186352; doi:10.7150/ijbs.93843)
Supplement: Supplementary file 1 — Supplementary figures and table. [file ijbsv20p3094s1.pdf]

Supplemental Figure 1. The DNFB induced AD model was successfully constructed

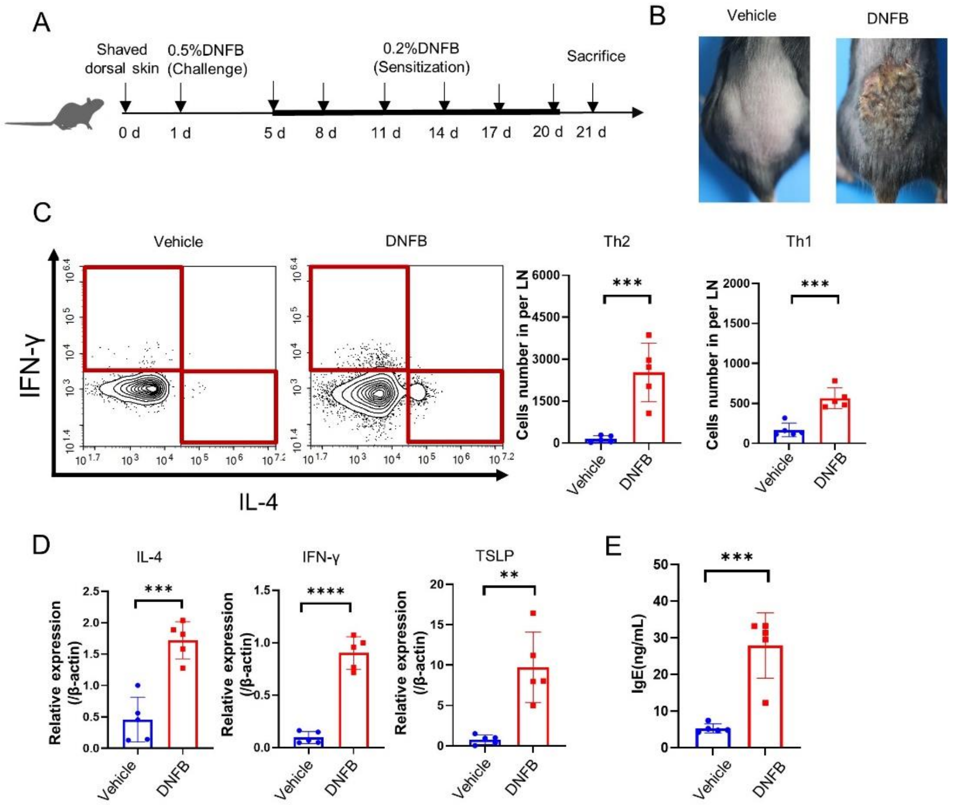

(A) Schematic diagram of the protocol for DNFB induced AD mouse model.

(B) Representative graph of skin in WT mice treated with DNFB or vehicle.

(C) Flow cytometry measurement the number of Th1 and Th2 cells differentiation in lymph nodes of WT mice (n=5) after treating with DNFB or vehicle. Mean ± SD.

(D) Relative expression of multifarious inflammation cytokines in WT mice (n=5) of AD-lesion or normal skin were quantified by RT-qPCR after DNFB or vehicle induced. Mean ± SD.

(E) Determination of IgE in serum of WT mice (n=5) by ELISA after DNFB or vehicle induced. Mean ± SD. Error bars represent the mean ± SD. ns, not significant; **$p < 0.01$; ***$p < 0.001$; ****$p < 0.0001$; p values were calculated using Student's t test.

Supplemental Figure 2. Construction and identification of the IL-38 keratinocyte-specific knockout mice

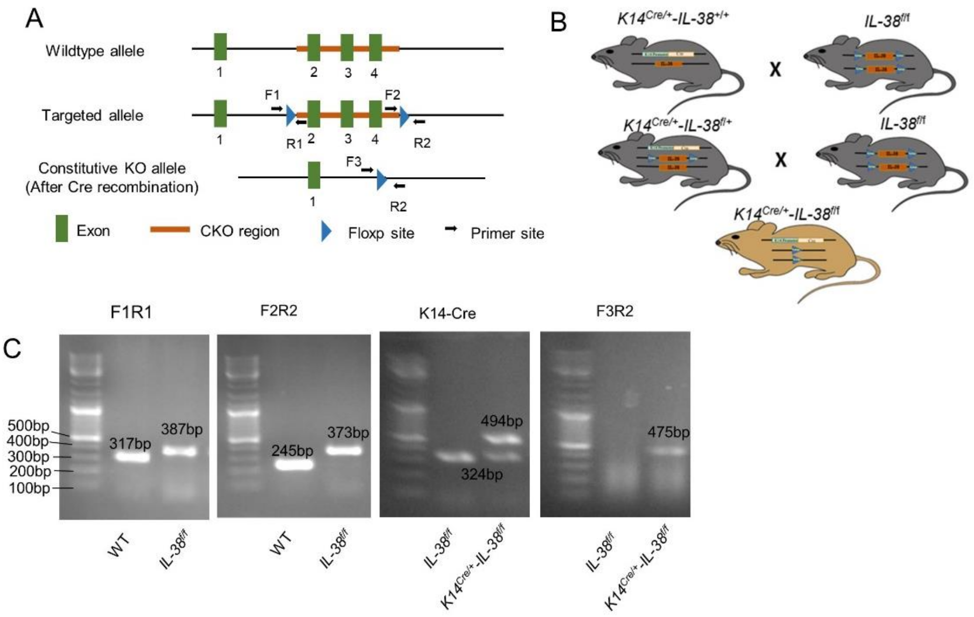

(A)Scheme of Cre/LoxP strategy for *IL-38* exons 2 to 4 excision.

(B)Generation of mice with IL-38 specifical deletion in keratinocytes (*K14$^{Cre/+}$-IL-38$^{f/f}$*) by breeding *IL-38$^{f/f}$* mice with *Krt14-Cre* transgenic mice(*K14$^{Cre/+}$*).

(C) Agarose gel electrophoresis of the PCR analysis of genomic IL-38 excision of mice. Primers F1R1 and F2R2 were used to identify mice containing the floxp site, followed by *Krt14-Cre* primers to detect the presence of the *Krt14-Cre* enzyme gene, and then F3R2 primers to detect the shearing of the *IL-38* gene in mice containing both the *Krt14-Cre* enzyme gene and the floxp site.

Supplemental Figure 3. The flow cytometry gating strategy for multifarious immune cells

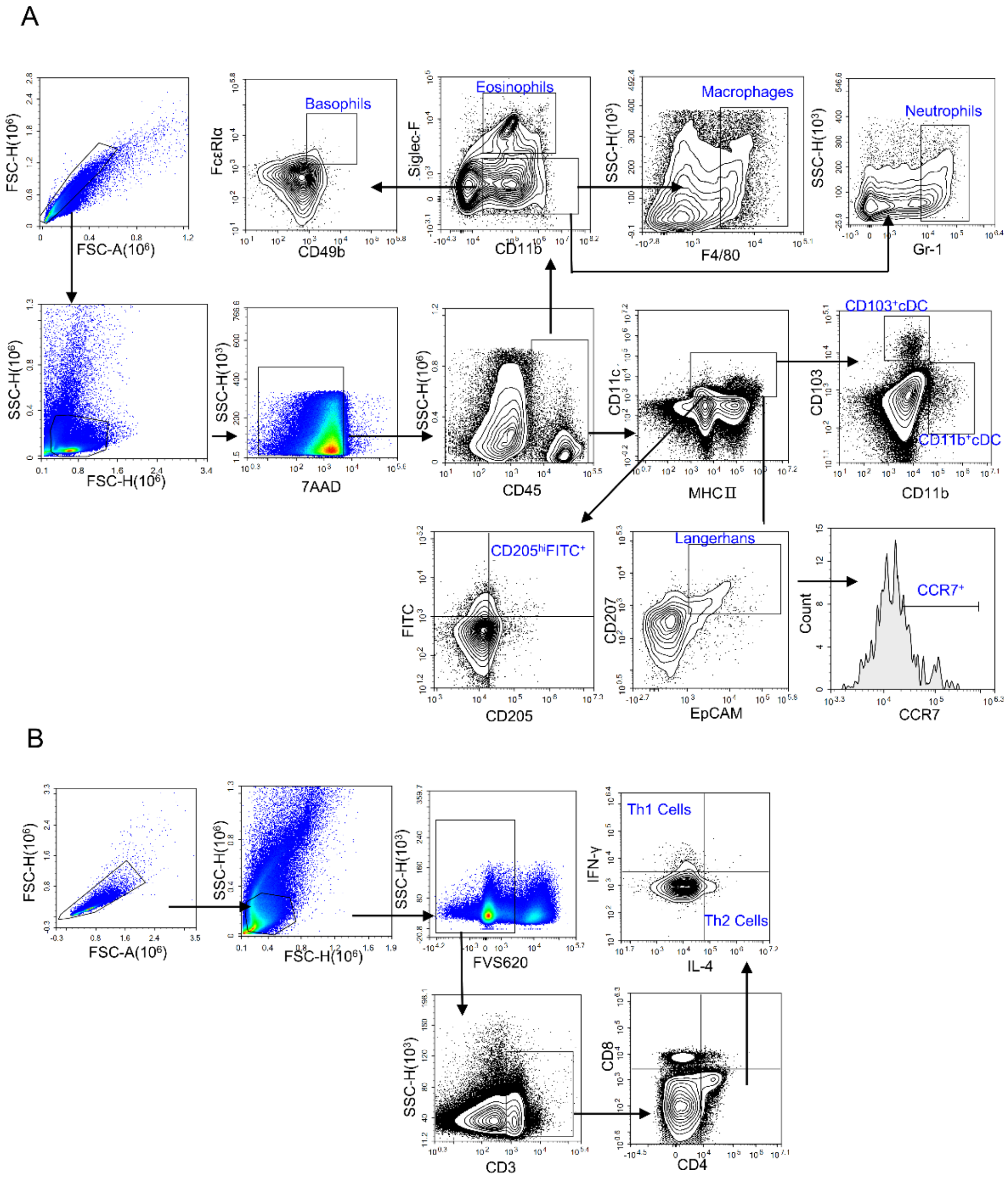

(A) Several types of immune cells about the flow cytometry gating strategy.

(B) The flow cytometry gating strategy of Th1 and Th2 cells differentiation from $CD4^{+}$T cells in lymph nodes.

Supplemental Figure 4. Subcutaneous injection of IL-38 promoted the DNFB-induced AD-like skin inflammation.

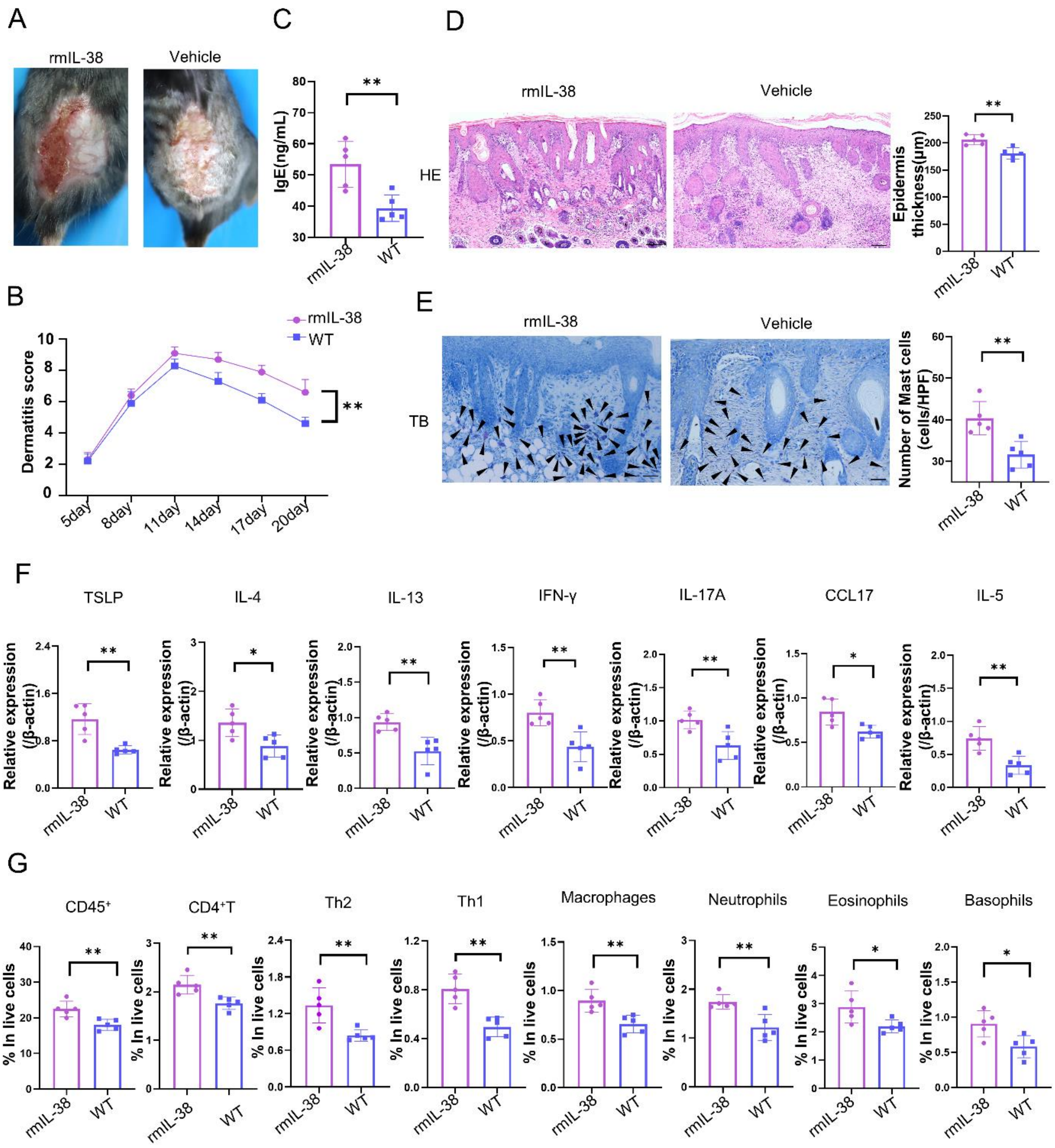

(A) Representative graph showing the status of dorsal skin lesions after subcutaneous injection of rmIL-38 or vehicle in DNFB-induced WT mice.

(B) The total dermatitis scores were assessed in AD-lesions after subcutaneous injection of rmIL-38 (n=5) or vehicle(n=5) in DNFB-induced WT mice.

(C) Determination of IgE in serum was assessed by ELISA after subcutaneous injection of rmIL-38 (n=5) or vehicle(n=5) with DNFB induction.

(D) H&E staining was performed to detect epidermal proliferation in the AD-lesion after subcutaneous injection of rmIL-38(n=5) or vehicle(n=5) with DNFB induction. Scale bars represent 100 μm.

(E) Toluidine blue staining (TB) was performed to measure alterations in the number of mast cells in the lesion area of mice treated with rmIL-38(n=5) or vehicle(n=5) after DNFB induction, arrows on the graph indicate mast cells. Scale bars represent 50 μm.
(F) Relative expression of various inflammatory cytokines was quantified by RT-qPCR in AD-lesions after subcutaneous injection of rmIL-38 (n=5) or vehicle(n=5) in DNFB-induced WT mice.
(G) Flow cytometry assay the proportion of infiltrating immune cells in the total number of live cells in AD-lesions after subcutaneous injection of rmIL-38 (n=5) or vehicle(n=5) in DNFB-induced WT mice..
Error bars represent the mean ± SD. $*p < 0.05$; $**p < 0.01$; p values were calculated using Student’s t test or One-way ANOVA and Two-way ANOVA.

Supplemental Figure 5

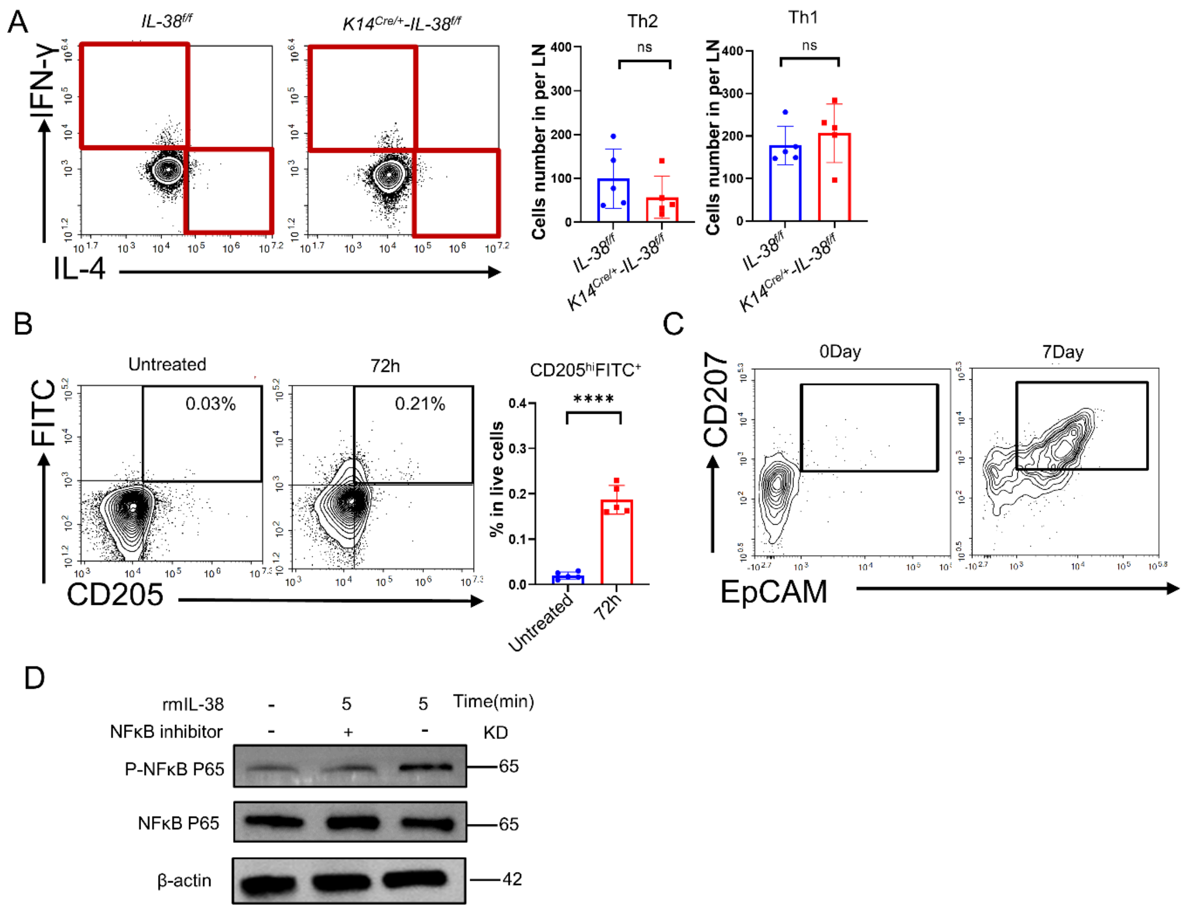

(A) Flow cytometry assays for the differentiation of Th1 and Th2 cells in the lymph nodes of *K14*$^{Cre/+}$*-IL-38*$^{f/f}$ (n=5) and *IL-38*$^{f/f}$ (n=5) mice at steady state.

(B)Kinetics of skin DC migration into the draining lymph node after FITC painting.C57BL/6 mice were shaved, depilated, and painted with 1% FITC solution prepared in a solution of inflammatory stimulating mixture of acetone and dibutyl phthalate (1:1, vol: vol) on the flank skin. Subsequently, the number of CD205^{hi}FITC^{+} migrating to lymph nodes in mice was detected and analyzed by flow cytometry. (n=5)

(C) Mouse bone marrow cells were isolated and differentiated into LCs by induction with the addition of 20ng/mL rmGM-CSF and 10ng/mL rhTGF-β for 7 days and then measured by flow cytometry.

Error bars represent the mean ± SD. ns, not significant; ****p < 0.0001; p values were calculated using Student's t test

(D) Mouse bone marrow primary cells were isolated and induced as BM-LCs, stimulated by adding rmIL-38 for 5min directly or NF-κB inhibitor QNZ pretreatment for 2h. Proteins were extracted after collecting cells and WB assayed for expression of NF-κB phosphorylation.

Supplemental Figure 6. Combined IRAK4/ NF-κB inhibitor attenuates DNFB-induced AD symptoms.

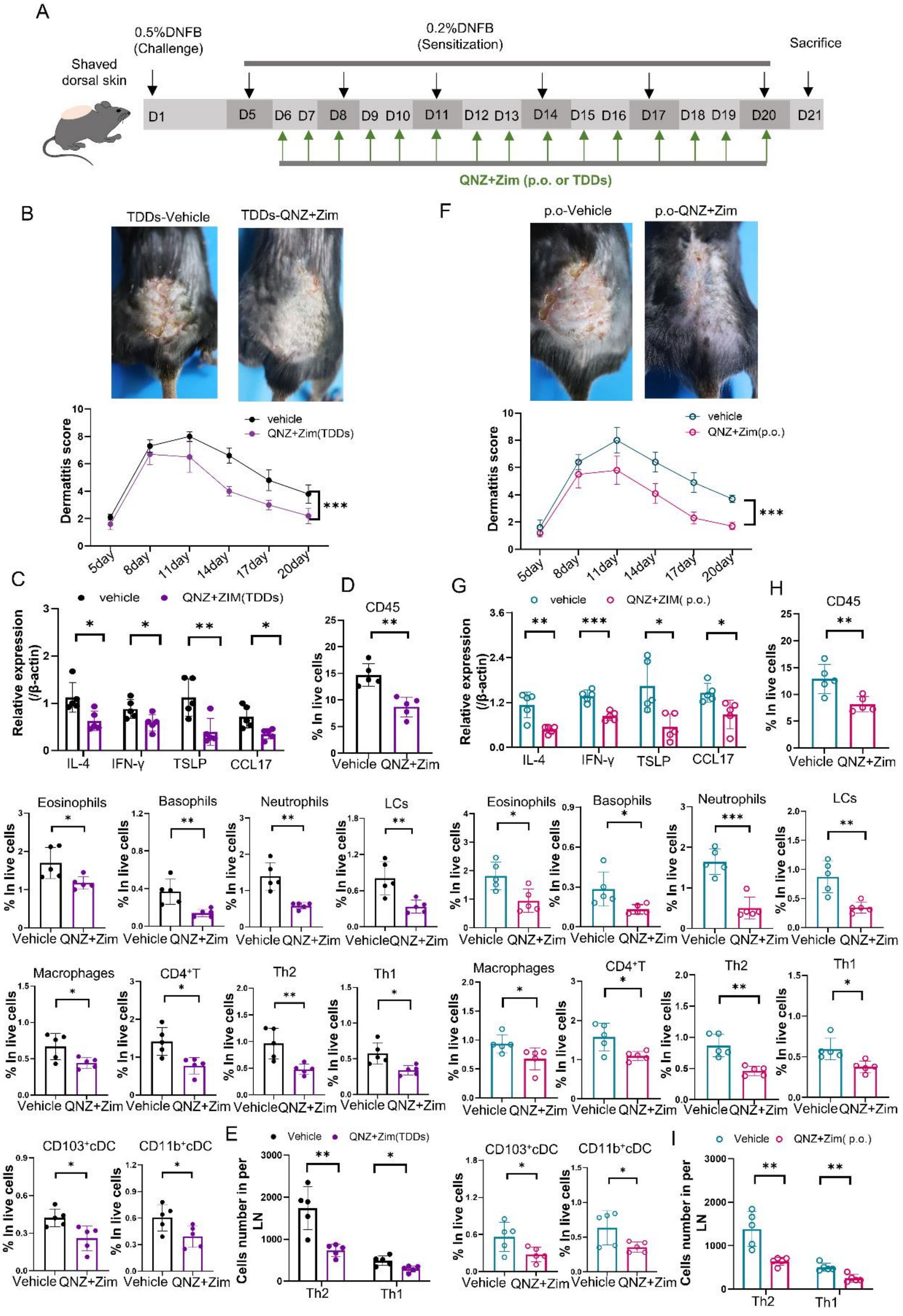

(A) The schematic of the scheduled treatment in DNFB-induced AD mode of wild-type mice by combination IRAK4 inhibitor Zimlovisertib and NF-κB inhibitor QNZ. (Zim, Zimlovisertib)

(B) Mice were treated by combination Zimlovisertib and QNZ or vehicle in AD-lesion after DNFB-induced by TDDs. The upper graph representative of dorsal skin lesions status, while the dermatitis scores in dorsal lesion areas in the lower (n=5). (TDDs, Transdermal drug delivery systems)

(C) Relative expression of multifarious inflammation cytokines on AD-lesion skin in mice which were treated with Zimlovisertib and QNZ (TDDs) or vehicle in AD-lesion after DNFB induced were quantified by RT-qPCR. (n=5).

(D) Flow cytometry assay the percentage of infiltrating immune cells in mice of lesion skin which were treated by combination Zimlovisertib and QNZ (TDDs) or vehicle in AD-lesion after DNF-induced. (n=5).

(E) Flow cytometry assay number of Th1 and Th2 cells differentiation in lymph nodes of mice treated by combination Zimlovisertib and QNZ(TDDs) or vehicle in AD-lesion after DNFB-induced. (n=5).

(F) Combination Zimlovisertib and QNZ or vehicle were given orally(p.o.) to mice after DNFB-induced. The upper graph representative of dorsal skin lesions status and the dermatitis scores of AD-lesion skin in the lower. (n=5).

(G) Relative expression of multifarious inflammation cytokines on AD-lesion skin in mice treated by combination Zimlovisertib and QNZ (p.o.) or vehicle after DNFB-induced. (n=5).

(H) Flow cytometry assay the percentage of infiltrating immune cells of lesion skin after treated by combination Zimlovisertib and QNZ (p.o.) or vehicle in DNFB induced mice. (n=5).

(I) Flow cytometry assay number of Th1 and Th2 cells differentiation in lymph nodes after treated with Zimlovisertib and QNZ (p.o.) or vehicle in DNFB induced mice. (n=5).

Error bars represent the mean ± SD. $^{*}p < 0.05$; $^{**}p < 0.01$; $^{***}p < 0.001$; p values were calculated using Student’s t test or One-way ANOVA and Two-way ANOVA.

Supplemental Table

Table 1 The primers of mice

Gene	Forward (5' to 3')	Reverse (5' to 3')
F1R1	TCACAGTTACTACATCTTCAGTTCC	CACCTTACTGGACAACCACATGGAG
F2R2	CCATGAAGCTCTGGCTAGACACCA	CACACCATAGCCCAGACATGCC
K14-Cre	TTCCTCAGGAGTGTCTTCGC	GTCCATGTCCTTCCTGAAGC
K14-Cre IPC	CTAGGCCACAGAATTGAAAGATCT	GTAGGTGGAAATTCTAGCATCATCC
β-actin	CCTCTATGCCAACACAGTGC	ACATCTGCTGGAAGGTGGAC
IL-38	AGCTTGGGATCTGCCTTCAG	CAGTATGGGTGGAGGGTTCAC
TSLP	ACGGATGGGGCTAACTTACAA	AGTCCTCGATTTGCTCGAACT
IL-4	GGCGCTGGACCTGTGGGTTG	CCGTGCATGGCGTCCCTTCTC
IFN-γ	ATGAACGCTACACACTGCATC	CCATCCTTTTGCCAGTTCCTC
IL-5	ATGGAGATTCCCATGAGCAC	GTCTCTCCTCGCCACACTTC
IL-17A	CTCAGACTACCTCAACCGTTCC	CATGTGGTGGTCCAGCTTTCC
IL-13	CATGGCCTCTGTAACCGCAA	TGGCGAAACAGTTGCTTTGTG
CCL17	CGAGAGTGCTGCCTGGATTACT	GGTCTGCACAGATGAGCTTGCC
CCL19	CATAAATTGGAGCTGGTGGCAG	AGGAGCCAAGTGCAAGTGAG
CCL21	AGGAAGAACCGGGAACCTC	AGGGCTGTGTCTGTTCAGTTCTC
